# The Risk of Infection by African Swine Fever Virus in European Swine Through Boar Movement and Legal Trade of Pigs and Pig Meat

**DOI:** 10.3389/fvets.2019.00486

**Published:** 2020-01-09

**Authors:** Rachel A. Taylor, Roberto Condoleo, Robin R. L. Simons, Paul Gale, Louise A. Kelly, Emma L. Snary

**Affiliations:** ^1^Department of Epidemiological Sciences, Animal and Plant Health Agency (APHA), Weybridge, United Kingdom; ^2^Istituto Zooprofilattico Sperimentale Lazio e Toscana “M. Aleandri”, Rome, Italy; ^3^Department of Mathematics and Statistics, University of Strathclyde, Glasgow, United Kingdom

**Keywords:** risk assessment, disease transmission, pork product, swine disease, wild boars, European Union (EU), disease hotspot, riskiest pathway

## Abstract

African swine fever (ASF) is currently spreading westwards throughout Europe and eastwards into China, with cases occurring in both wild boar and domestic pigs. A generic risk assessment framework is used to determine the probability of first infection with ASF virus (ASFV) at a fine spatial scale across European Union Member States. The framework aims to assist risk managers across Europe with their ASF surveillance and intervention activities. Performing the risk assessment at a fine spatial scale allows for hot-spot surveillance, which can aid risk managers by directing surveillance or intervention resources at those areas or pathways deemed most at risk, and hence enables prioritization of limited resources. We use 2018 cases of ASF to estimate prevalence of the disease in both wild boar and pig populations and compute the risk of initial infection for 2019 at a 100 km^2^ cell resolution via three potential pathways: legal trade in live pigs, natural movement of wild boar, and legal trade in pig meat products. We consider the number of pigs, boar and amount of pig meat entering our area of interest, the prevalence of the disease in the origin country, the probability of exposure of susceptible pigs or boar in the area of interest to introduced infected pigs, boar, or meat from an infected pig, and the probability of transmission to susceptible animals. We provide maps across Europe indicating regions at highest risk of initial infection. Results indicate that the risk of ASF in 2019 was predominantly focused on those regions which already had numerous cases in 2018 (Poland, Lithuania, Hungary, Romania, and Latvia). The riskiest pathway for ASFV transmission to pigs was the movement of wild boar for Eastern European countries and legal trade of pigs for Western European countries. New infections are more likely to occur in wild boar rather than pigs, for both the pig meat and wild boar movement pathways. Our results provide an opportunity to focus surveillance activities and thus increase our ability to detect ASF introductions earlier, a necessary requirement if we are to successfully control the spread of this devastating disease for the pig industry.

## Introduction

African Swine Fever (ASF) is an infectious disease of several members of the *suidae* family that is caused by African swine fever virus (ASFV) belonging to the Asfarviridae family (1). Animals can become infected with ASFV via nasal, oral, subcutaneous, or ocular penetration, and once infected they will shed the virus into the environment (2), and even after death their carcasses can continue to contribute to virus dissemination since the virus can persist in blood and tissues for prolonged periods. It has been demonstrated that the virus can remain viable in feces and urine up to 8 and 15 days, respectively, (3) and for weeks in pork meat and processed products (4–8). There is also the possibility that ASFV can spread via soft ticks, which is the predominant method of spread between warthogs, bush pigs, and wild pigs in the disease origin area in sub-Sahara Africa, although they are not a requirement for disease spread (1). Thus, ASFV has a high capacity for transmission by both direct and indirect contact with infected animals, or via the environment, vectors, and animal products.

ASF is recognized as one of the most important and dreaded diseases of pigs for several reasons. Firstly, it can spread to pigs in disease-naïve areas through numerous different entry routes such as trade in live animals and animal products, wild animal migrations, fomites, vehicles, and vector movements (9, 10). This makes it particularly challenging to implement effective preventive measures in order to minimize the risk of incursion of the disease. Secondly, until now, no vaccine has been available for ASF (10). Thirdly, if introduction of ASF does occur in a region, the disease can have a devastating impact on the swine sector as it can result in huge losses (10). Some viral strains demonstrate a high virulence potential and can effect a lethality rate near 100% (11). Once the introduction of the disease in a region is confirmed, culling of sick animals and restrictions for live animal and animal product trade follow in order to limit the spread of the disease to neighboring areas or other countries; these mitigation measures lead to further economic damage for farmers and food producers. Finally, management and eradication of the disease in areas with new incursions or endemic areas is difficult, resource-intensive, and costly due to the infectivity durability of the virus in the environment, the many transmission pathways and the potential establishment of a wild animal reservoir population, which could be supported by competent vectors (if present) (1). Therefore, effective methods to help decision makers decide where to focus limited surveillance resources are very useful and can help to reduce the economic impact of an ASF outbreak.

The epidemiological situation of ASF has changed rapidly over the last few decades. Historically, the disease was mainly present in the African swine population but, after entering Portugal in 1957 likely due to swill feeding with contaminated food waste, it became endemic in the Iberian peninsula and several linked outbreaks were observed in Italy, France, Malta, Belgium, and the Netherlands during the following years (12). Apart from Sardinia, where the disease continues to persist until now, ASF was considered completely eradicated from Europe in 1995. However, in 2007, outbreaks were reported in domestic and wild boars in Georgia, the first time outside of sub-Saharan Africa in many years (1). The virus introduction was probably caused by the import of contaminated meat from Madagascar or Mozambique (12). Despite the effort to contain and eradicate the disease, ASF spread progressively to other countries in the Caucasian region such as Armenia, Azerbaijan, and the Russian Federation in 2007–2008 (12, 13). Migration of infected wild boars, trade of infected animals and derived meat products, poor biosecurity measures and delayed notification by small farmers seem to be the most important factors that hampered the disease eradication and lead to a constant spread toward the European Union (EU) during the following years (14, 15). New outbreaks concerning domesticated and wild pigs were reported in Ukraine (2012), Belarus (2013), Poland and Baltic countries (2014) (16). EU Member States (MS) responded to this rapid escalation of the disease situation by intensifying biosafety mitigation measures and setting up specific surveillance programmes and information campaigns for veterinarians, farmers, and travelers (9). However, until now (May 2019), ASF is still present in the mentioned countries and outbreaks in pigs or wild boars have also been notified by the Czech Republic, Hungary, Bulgaria, and Romania (12, 16). Several ASFV infected wild boars were also found in Belgium confirming that ASF is able to make large geographic jumps (15). In addition, since 2017 the disease began to progress rapidly in an easterly direction, with the Russian Federation registering new cases in East Siberia followed by China in 2018 and Mongolia, Vietnam, and Cambodia in 2019 (17).

Direct contact between sick and healthy animals is one of the most evident ways of virus transmission considering that saliva, urine and feces are heavily contaminated (18). Although animal health legislation of all European countries bans live animal trade from high-risk areas, the absence of clinical signs during the latent period makes the early identification (and notification) of new outbreaks in ASF-free regions difficult as well as the detection of infected pigs by the border authorities of the destination country. As such, there can be a fairly large time window at the start of an ASF outbreak in a region where no one is aware the disease is circulating in the pig population and thus infected pigs may be traded. Another prominent cause of incursion of ASFV in European ASF-free areas is by import of pork products derived from infected animals (2). If the disease is not detected by Local Authorities at farm or abattoir level, infected pigs can be slaughtered and their contaminated carcasses used for fresh or processed pork products. Swill feeding is illegal in most countries around the world, including the EU, but despite this, some pigs raised in backyard or free-ranging small farms are fed with untreated food leftovers or catering waste (18). The prolonged survival of the virus in edible tissues and the low infectious dose required to infect animals make this transmission pathway particularly relevant, as proven by several outbreaks in recent years (19). Even if waste pork products are properly disposed of, wild boar are still potentially able to get access to landfills or waste bins, become infected after contacting/ingesting the contaminated food, and transmit the virus to the local animal population. Wild boar can play an important epidemiological role not only in this transmission pathway but they can also be responsible for transboundary spread of the disease due to their natural dispersal ecology in search of new territory (20). Indeed, although wild boars normally remain close to their natal home range, studies reported that they are capable of covering long distances (up to 250 km) (21). A new outbreak can occur when infected wild boar are able to gain access to farms and contact susceptible domestic pigs, such as those on farms with poor biosecurity measures. As an example, the initial infection of the Lithuanian pig population with ASF is believed to be due to movements of infected wild boars from Belarus (19, 22).

Due to the importance of ASF and the recent incursion into Europe, several risk assessments have been performed in order to estimate the likelihood of introduction of ASF into Europe or specific European countries, using qualitative, semi-quantitative and quantitative models. In 2010, the European Food Safety Authority (EFSA) evaluated the risk of endemicity of ASF in the Caucasus region and the consequent risk of release into Europe through several potential routes (13). A few years later, the same qualitative model, which was based on expert elicitation, was used to update the results given new epidemiological and experimental evidence (23). Recently, two similar semi-quantitative methodologies, which rely on expert knowledge, were developed to assess the ASF risk, via multiple pathways, to Belgium (24) and Finland (25). Furthermore, semi-quantitative frameworks, which score the risk of ASFV release in the EU, have been published for a single route of disease entry such as wild boar movements (26, 27), illegal importation of pork and pork products (28) and transport-associated routes (29). Regarding the quantitative risk models, only a few have been developed until now. Mur et al. (30) estimated the probability of ASFV entry for each country of the EU through the import of live pigs and, a few years later, the same group presented a risk assessment considering multiple ASFV entry pathways of high relevance for transmission, although some of them were still modeled adopting a semi-quantitative approach (31). Recently, Lange et al. (32) developed a mechanistic model to simulate the spread of the disease in wild boar populations in Europe and subsequently to assess the impact of control measures, presence of natural, or artificial barriers and management strategies of wild animal populations implemented by affected EU MSs. Lastly, Simons et al. (33) assessed the risk of entry of ASF into EU MSs using a quantitative model for a number of pathways, although did not consider whether this could lead to infection in susceptible pigs or wild boar in those countries.

Apart from Lange et al. (32), all of the models listed above, whether qualitative or quantitative, assessed the risk for a single or multiple EU countries at a country level. Furthermore, some of the models assessed the risk of entry only and did not consider the transmission to susceptible pig or wild boar populations. Only one of the models (33) was able to quantitatively assess the incursion risk by multiple pathways, however, due to only assessing risk of entry, it was not possible to compare pathways in that model to indicate which pathway was of greatest risk. In this study, we adapt a generic risk assessment framework (34) to assess quantitatively the risk of infection with ASFV in domestic pigs or wild boar across Europe at a fine spatial scale (100 km^2^ cells) via multiple pathways, namely trade in live pigs, trade in pig meat products, and movement of wild boar. We create risk maps for 2019 of the probability of infection in pigs and boar for each pathway and for all pathways combined, in order to identify hotspots of ASFV incursions in the EU, and the pathways of most importance in each area.

## Materials and Methods

We assess the probability of at least one infection in boar or pigs through three pathways of transmission within Europe, namely: legal trade of pigs, legal trade of pig meat, and movement of wild boar. We acknowledge that other pathways may be important for transmission of ASFV, such as legal movement of meat within the EU via travelers, but could not find data of sufficient detail or quality to parametrize these with enough certainty (2, 9). The wild boar pathway is assessed for the whole of Europe, whereas the other two pathways are restricted to EU MSs only due to lack of data in other European countries. The approach is stochastic and applies the framework outlined in Taylor et al. (34). The risk assessment framework is outlined in brief below and we highlight how it is followed for each of the pathways. The risk of ASF is assessed through these three pathways separately and combined into one overall risk at a spatial scale of 100 km^2^ raster cells. The risk assessment uses reported cases in 2018 in order to predict the risk of ASF infection in 2019.

### The Generic Risk Assessment Framework

**Figure 1:**
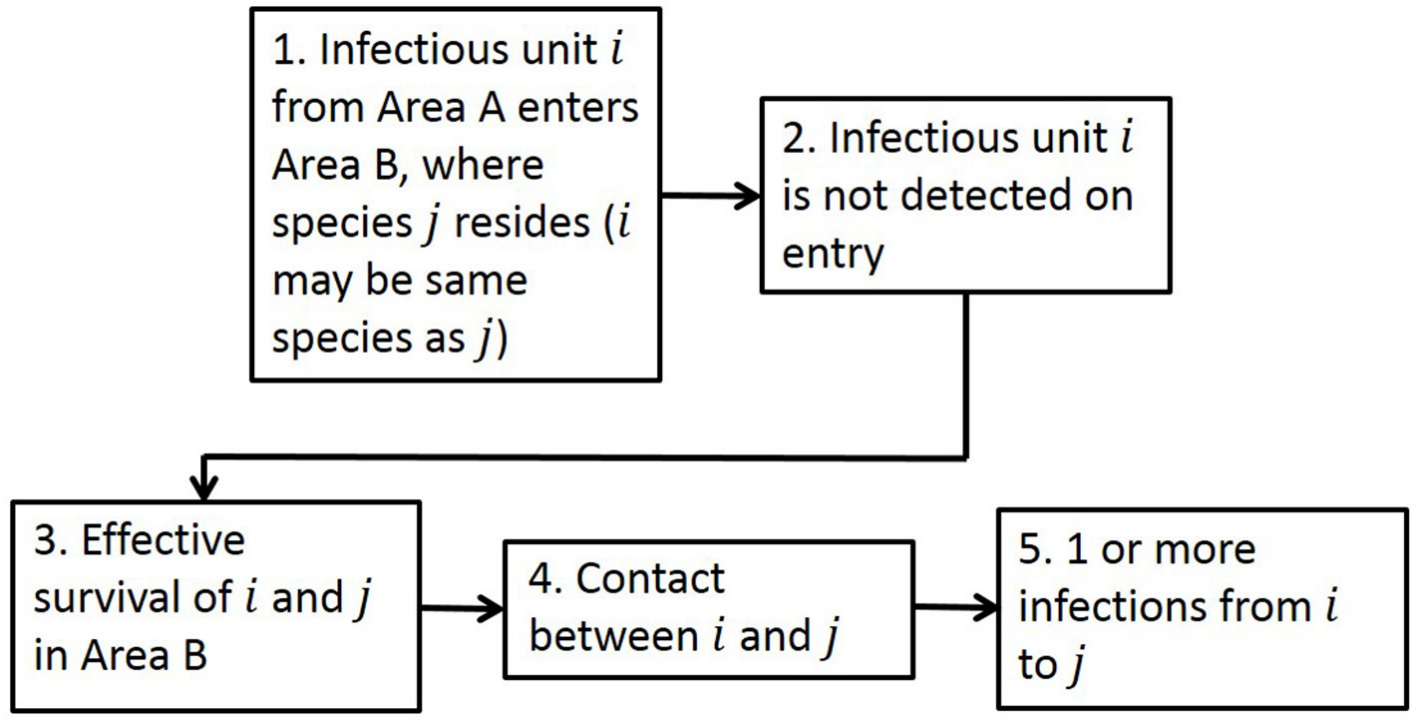
The generic framework for calculating risk of infection spatially, as seen in Taylor et al. (34). There are five steps in the framework: Entry, Detection, Survival, Contact, and Transmission to address the risk question “What is the risk of infection of a pathogen in Area B due to the presence of that pathogen in Area A?” The term “unit” in Step 1 addresses the fact that it could be infected animals or products or contaminated items entering.

The generic framework for performing quantitative spatial assessments of risk of infection is designed to be suitable for many disease entry pathways (Figure 1), with the aim of answering the risk question “What is the risk of infection of a pathogen in Area B due to the presence of that pathogen in Area A?”

Adopting this framework, we calculate the risk of initial infection with ASFV via a single pathway by estimating how many infectious animals/products (depending on the entry pathway) will enter a new Area B given the disease is present in Area A, whether detection of those animals/products would occur, the probability of survival of the animal and pathogen, and the rate of contact and transmission to susceptible animals in Area B. In this risk assessment for ASF, Area B is defined to be EU MSs and Area A is the whole world. We compute the risk assessment at a 100 km^2^ cell level in Europe by first calculating the number of infected units entering cell *c* of Area B, *I*_*k*_(*c*), as:

(1) $$\begin{array}{r} I_{k}\left(c\right)\sim Bin\left(N_{k}\left(c\right),p_{k}\right), \end{array}$$

where *k* is a region of Area A (defined for each pathway), *N*_*k*_(*c*) is the number of units (pigs/boar/pig meat) moving from region *k* to cell *c* and *p*_*k*_ is the prevalence in the relevant species in region *k*. We thus define $I\left(c\right)=\;\underset{k}{\sum }I_{k}\left(c\right)$ as the total number of infected units entering cell *c* from all of Area A. For the trade pathways, we define the regions of Area A to be countries around the world. For the wild boar movement pathway, we define the regions of Area A to be 100 km^2^ cells in which we estimate prevalence to be non-zero, based on reported cases in Europe.

For the detection step, in this ASF case study, we assume there is no detection of infected animals or products in the trade or movement pathways. The only relevant EU decree regarding movement of pigs or pig meat involves a restriction on trade from ASFV infection zones once cases are detected, and we account for this in the prevalence estimates. Otherwise, any testing of pigs or pig meat is voluntary and the exact procedures may vary widely by country. We therefore assume no inspection or testing of traded pigs and pig meat occurs, and treat this as a worst-case scenario. However, we do perform a scenario analysis with detection included, with method and results provided in the Supplementary Info S3.

We use the disease metric *R*_0_ to describe the survival of the pathogen/animal, as well as contact and transmission between the infectious units and susceptible pigs and wild boar. *R*_0_ is a measure of the expected number of new infections that would occur if one infectious unit were to enter a susceptible population. Our equation for *R*_0_(*c*) includes information on the susceptible populations at risk in cell *c*, the pathway of entry, the transmission between infected and susceptible animals and the duration of infection. Each parameter from *R*_0_(*c*) is drawn from a distribution of likely values. The number of initial infections, *N*_*I*_(*c*), in cell *c* is then calculated by *I*(*c*) draws from a Poisson distribution with mean given by *R*_0_(*c*):

$$\begin{array}{l} {\;N}_{I}\left(c\right)=\overset{I\left(c\right)}{\underset{1}{\sum }}\xi \\ \xi \sim \;Pois\left(R_{0}\left(c\right)\right). \end{array}$$

Each infected animal entering cell *c* will in turn infect an average of *R*_0_(*c*) susceptible animals. We represent this by a Poisson distribution with mean *R*_0_(*c*), as this distribution simulates the number of events that can occur given the expected number, and ensures a non-negative integer value is returned. There are *I*(*c*) infected units entering cell *c*, each with the same Poisson distribution, which produces the summation, to give *N*_*I*_(*c*) new infections overall. The probability *R*_*I,X,p*_(*c*) or *R*_*I,X,b*_(*c*) that at least one susceptible animal would become infected in cell *c* due to entry via a single pathway *X* is then given by the proportion of the simulations where infection occurs in a susceptible pig or boar, respectively. We outline in greater detail below how we compute each step of the risk assessment for each of the pathways below. Within the model, we use the Poisson distribution, as outlined above, or the Binomial distribution to simulate the expected number of successful events that occur if each event has the same probability of success.

### Legal Trade of Live Pigs

The legal trade of live pigs is the easiest to implement under the framework outlined above as there is good data availability for parameters for each step in the pathway. For the entry of infectious animals, we use trade data from TRACES (35) to determine the total number of animals (*N*_*k*_(*c*)) moving from each region *k* of Area A to each cell *c* of Area B and input this into Equation (1). The TRACES data includes a post-code for the destination of the trade, the country of origin and total number of animals moving in 1 year from that country to that post-code destination. We convert the destination post-codes to latitude and longitude values to find the 100 km^2^ cell of entry. We calculate prevalence at a country level to coincide with the TRACES data on origin of trade. For prevalence data in each country of the world, we use output from the model outlined in Simons et al. (36), which uses OIE data on recent reported cases of ASF (excluding cases in Sardinia due to successful containment on the island) to calculate prevalence in each country of the world. This model for prevalence incorporates under-reporting, trade restrictions within infected zones and the length of latent period of the disease to compute an accurate prevalence estimate. We assume that all animals are destined for farms. Therefore, we assess the survival, contact and transmission rates assuming contact with susceptible pigs on a farm. To calculate the survival, contact and transmission between imported pigs and susceptible pigs on a farm, we use the following equation for *R*_0_:

$$\begin{array}{l} R_{0}=\frac{\gamma \beta S\left(c\right)}{r}. \end{array}$$

In this equation, γ is the contact rate between pigs, β is the probability of transmission given contact between pigs, *S*(*c*) is the number of susceptible pigs that the animal could have contact with and *r* is the rate of an infected pig dying from or recovering and no longer shedding ASFV. We assume no contact with wild boar in this pathway. For further details of how this pathway is computed, see Supplementary Info S1 and Taylor et al. (34) in which a case study for the live animal trade pathway was performed for Lumpy skin disease.

### Movement of Wild Boar

As there are no datasets on wild boar movement between countries, never mind between 100 km^2^ cells, we instead use a model of boar movement at the cell level. This is based on boar ecology, such as which age groups and gender tend to undergo dispersal events compared to home range movement and the reason for movement. The model for movement of boar is adapted from Simons et al. (33) and is explained fully in Taylor et al. (37); we outline the key steps here. The model uses data on boar abundance and the habitat suitability of Europe at a cell level for boar (38). We include two types of boar movement—within a home range and long-range dispersal. When performing home range movement, the boar traverses the whole of its home range area. For long-range dispersal, we fix a total number of steps that each dispersing boar will perform, based on maximum distances of boar dispersal and our cell size. In order to determine the direction of boar movement from each cell we use the habitat suitability score for each cell as a proxy for deciding where boar would want to move to. We calculate the ratio between the habitat suitability in each neighboring cell and the overall habitat suitability of all the neighboring cells and use that to determine the probability of the boar moving to each cell. We assume one dispersal event each year and assume that a dispersing boar spends the rest of the year performing home-range movement.

We calculate *N*_*k*_(*c*), the number of boar moving from all cells (*k*) in Area A which have non-zero prevalence to cells (*c*) in Area B, by combining the probability of moving to each cell and the total number of boar in the origin cell, using an abundance map of boar across Europe (38). Then to determine how many infected boar enter each cell *c* in Area B using Equation (1), we estimate prevalence in wild boar for each origin region *k*, which in this case are also 100 km^2^ cells, using the locations of reported ASFV cases in wild boar, an under-reporting factor to account for infected boar carcasses not being found or reported, and the abundance of boar in each cell.

We calculate the potential transmission of ASFV from these infected boar to susceptible boar and pigs in the destination cell using two separate equations for *R*_0_ for transmission to boar and pigs. For wild boar contact with pigs, we use a similar formula to the *R*_0_ for live pig trade, in which we include the number of susceptible pigs in the area at risk, the length of the infectious period in live boar, the contact rate between boar and pigs and the probability of transmission given contact. We adapt the equation for *R*_0_ for boar by including two additional factors—group dynamics and contact with carcasses. As wild boar primarily live in matrilineal groups (39), we use different contact rates for within and between group contact. ASFV is both highly virulent with almost 100% mortality and highly persistent, such that carcasses can remain contaminated for a long time. Thus, we include the possibility that a susceptible boar could become infected from an infected boar carcass, by considering data on contact rates between live boar and boar carcasses, the probability of transmission via such contact and the length of time the boar carcass is available for contact. The equations for *R*_0_ are included in Supplementary Info S1 and further explanation is provided in Taylor et al. (37).

### Legal Trade of Pig Meat Products

There are datasets available on the amount of legally traded pig meat products, such as from Comext (40) which gives information on the amount (in tons) of pig meat products that are imported into each EU MS from both within and outside of the EU. Similar to the legal trade of pigs pathway, we use output from the model reported in Simons et al. (36) to estimate the prevalence of ASFV in pig meat products at time of slaughter in each country of the world. When calculating the entry of pig meat products from infected pigs, we use the same Binomial formula as outlined above but now use volume of product instead of number of animals—our unit in Figure 1 is now grams of pig meat products. We also have to take into account the many different product types that fall under the category of pig meat. The Comext data are split into different product types by a product code, representing the different products that are traded and the different processes each of the products may undergo. We simplify this data into 12 categories for product type, based on similarities of product composition, and five processes that the product may undergo, which leads to 21 product types overall (since not all product types will undergo all types of processes). See Supplementary Info S1 for a final list of all product types and processes. We keep these separate at this stage and estimate the amount of product from infected pigs for each of the 21 product types entering each EU MS.

**Figure 2:**
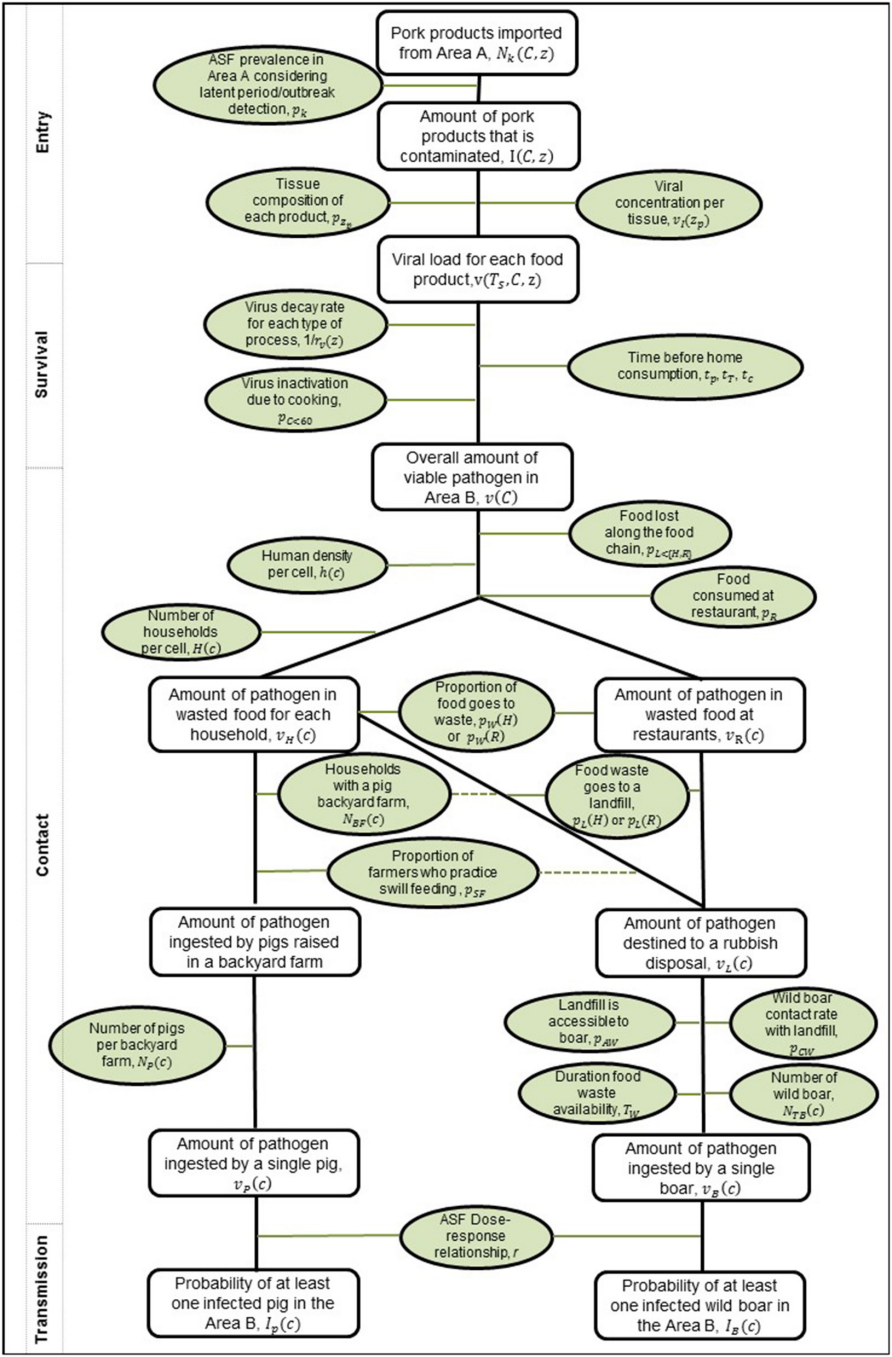
Risk pathway for the risk of ASFV infection in pigs and wild boar via the legal trade of meat products pathway. Probabilities or inputs considered for each step are outlined in green ovals, and the outputs along the pathway as rectangles. On the left, the boxes indicate how this framework fits within the more generic framework in Figure 1. The dashed lines indicate that the converse to the statement can also lead to meat products at a rubbish disposal.

As the legal trade in pig meat products is primarily for human consumption, this pathway focuses on estimating the probability that this meat for human consumption could end up inadvertently being contacted or consumed by domestic pigs or wild boar. We assume that wild boar can be in contact with food waste through landfill and other locations (e.g., waste bins outside households or in parks, nature reserves etc.) which may contain pig meat waste products. For domestic pigs we assume that biosecurity levels on commercial farms across Europe are high and so the probability of potential contact with imported pig meat is negligible and therefore it was decided it was not necessary to consider this further. However, for backyard pig farms we consider the possibility of contact due to illegal swill feeding. Due to the need to consider how pig meat products could end up in waste or being swill-fed to a backyard pig, there are a few additional considerations in the contact, survival, and transmission steps of the pathway. We include a more detailed version of the generic risk pathway for the food pathway (Figure 2) and describe these additional steps below.

#### Entry

The Binomial Equation (1) is used to compute the entry of product from infected animals by combining the total amount in grams of each product traded from the country of origin to the destination country and the prevalence in the country of origin. Similar to the live pig trade pathway, the prevalence at a country level is estimated using the method outlined in Simons et al. (33). We assume each gram of pig meat product from each country has the same probability of being derived from an infected pig. As above, we sum over all source regions to produce the total amount of infected product in grams entering each destination country *C*, for each product type *z*, which we denote by *I*(*C,z*).

#### Viral Load

For ease of computation regarding contact, survival and transmission, we switch at this point to perform our computations using the total viral load entering each country rather than the amount of infected product. We calculate *v*(*T*_*S*_, *C, z*), the initial viral load in product *z* for all grams of product *z* destined for country *C* at time of slaughter (*T*_*S*_). The viral load at time *T*_*S*_ will be higher than the viral load at time of entry to country *C*, but we include this decay over time in the survival step instead. As we do not include detection on entry, the amount of infected product on entry is the same at time of slaughter and time of entry. The initial viral load at time *T*_*S*_ is based upon the average composition of each product type from different animal tissues (namely meat, fat, offal, skin, and bone) and the viral load concentration in infected tissues. See Supplementary Info S1 for the proportions for each product type *z* split into the component tissues. We use estimates of an average viral load concentration in each tissue, from experimental data, assuming that the pig was slaughtered before clinical signs appeared (it was assumed that any pig showing clinical signs would be immediately removed from the food chain) and the virus concentration follows an exponential relationship from the day of infection until the viremia peak and decreases similarly until the day the virus is not detectable. Thus, the initial viral load is calculated as

$$\begin{array}{l} v\left(T_{S},\;C,\;z\right)=\;\underset{z_{p}}{\sum }p_{z_{p}}I\left(C,\;z\right)10^{v_{I}\left(z_{p}\right)}, \end{array}$$

where *z*_*p*_ represents the different tissues in product *z*, *p*_*z*_*p*__ is the proportion of product *z* that is composed of tissue *z*_*p*_ and *v*_*I*_(*z*_*p*_) is the initial viral load concentration in tissue *z*_*p*_.

#### Survival

We consider three steps when estimating the survival of the virus in each product type: the survival of virus during animal slaughter and meat processing, during transport time and during cooking. Processing occurs in the origin country, transport is between countries, and the cooking process for any raw meat imported occurs in the destination country *C*. Thus, this survival step estimates how the viral load will change from time of slaughter, *T*_*S*_, to the time of consumption of the product, irrespective of whether this product will end up being in contact with pigs or boar.

Different processes that may be applied to pig meat products include salting, drying, smoking, and being chilled or frozen. For virus survival during processing, we determine the remaining viral load in each product matrix after processing by assuming an exponential decay of viral load, using data on the decay rate in each tissue, the process that is undergone for that product type and the length of time that process will take. The viral decay rate, *r*_*v*_(*z*) depends on the product type *z*, as it is calculated based upon the maximum virus survival time in products that have undergone different processes. For estimating the decay due to processes, we did not include the fact that the processes themselves may reduce the viral load further, we only included the time taken for a process to occur. For some products, it is possible that multiple processes are applied, however, these processes may or may not be undergone simultaneously. Therefore, for these, we assume the process that takes the least amount of time as this is a worst-case assumption for amount of remaining viral load. Therefore, the viral load after time of processing (*T*_*p*_) of product type *z* destined for country *C*, *v*(*T*_*p*_, *C, z*), is

$$\begin{array}{l} v\left(T_{p},\;C,\;z\right)=v\left(T_{S},\;C,\;z\right){\left(1-r_{v}\left(z\right)\right)}^{t_{p}}, \end{array}$$

where *t*_*p*_ is the assumed time taken to undergo the process.

To estimate the viral load after transport (*T*_*T*_), we estimated the average time taken for transport between different countries. This was based on the distance between the country of origin of the trade and the destination country, the speed of two transport methods—fast (e.g., flight) or slow (e.g., ship/rail/lorry) transport between the countries, and the proportion of trade that would occur via fast or slow transport. For further details of how the average time, *t*_*T*_(*C*), for transport to country *C* was estimated, see Supplementary Info S1. Therefore, the viral load after transport *v*(*T*_*T*_, *C, z*), is calculated as:

$$\begin{array}{l} v\left(T_{T},\;C,\;z\right)=v\left(T_{p},\;C,\;z\right){\left(1-r_{v}\left(z\right)\right)}^{t_{T}\left(C\right)}. \end{array}$$

Lastly, we included virus survival during cooking in the destination country *C*. We assumed that products which had been salted, smoked, or dried would not undergo a cooking process but all other product types would. ASF virus is killed if food is cooked to 60°C for at least 10 min (41), and hence to determine the viral load remaining after cooking (*T*_*C*_), we apply the Binomial equation to the viral load after transport (for relevant products *z*) and the probability that the food is not cooked to at least 60°C, *p*_*C* < 60_.

$$\begin{array}{l} v\left(T_{C},\;C,\;z\right)\sim Bin\left(v\left(T_{T},\;C,\;z\right){,p}_{C<60}\right). \end{array}$$

Therefore, *v*(*T*_*C*_, *C, z*) is the final viral load in product *z* at time of consumption, based upon the decay of viral load from time of slaughter due to processing, transport time, and cooking. The total viral load in country *C* at time of consumption is then calculated by summing over all product types:

$$\begin{array}{l} v\left(C\right)=\;\underset{z}{\sum }v\left(T_{C},\;C,\;z\right). \end{array}$$

#### Contact

There are multiple steps to consider when asking how meat from an infected animal entering each EU MS would potentially have contact with susceptible pigs or wild boar. The Comext dataset on trade only indicates the destination country of the trade and does not give any finer spatial resolution. As the pig meat is intended for human consumption, we distribute the infected pig meat around the country based on human density in each EU MS at a 100 km^2^ cell level. Therefore, if in one cell in a country there lives 1% of the population of that country, then 1% of the infected pig meat entering the country will go to that cell. Therefore, the viral load entering each cell *c*, *v*(*c*), in country *C* is

$$\begin{array}{l} v\left(c\right)=v\left(C\right)\frac{h\left(c\right)}{h\left(C\right)}, \end{array}$$

where *h*(*c*) is the abundance of humans in cell *c* and *h*(*C*) is the abundance of humans in country *C*.

We assume that pig meat will have one of two destinations—a non-household setting (e.g., in restaurants or by food producers; hereafter referred to as restaurants) or a household. A small proportion of food is lost along the food chain, for example during distribution, prior to reaching a restaurant or household (*p*_*L*<{*H,R*}_), and then we split the total amount of infected meat by whether it will go to a restaurant (*R*) or a household (*H*). As pigs and boar are unintended recipients of the pig meat, we estimate how much meat they could be in contact with by estimating how much of the pig meat is wasted. Therefore, the total viral load in each cell that is wasted at a restaurant is estimated as

$$v_{R}(c)=\;p_{W}(R)p_{R}(1-p_{L<\left\{H,R\right\}})v\left(c\right),$$

where *p*_*R*_ is the proportion of pig meat that goes to a restaurant and *p*_*W*_(*R*) is the proportion of food wasted at a restaurant. We perform a similar calculation for the amount of viral load remaining if food goes to a household. As each backyard farm will be connected to a single household, we estimate the amount of viral load at a household, *v*_*H*_(*c*), that is subsequently wasted, as

$$v_{H}(c)=p_{W}(H)\frac{p_{H}(1-p_{L<\left\{H,R\right\}})v(c)}{H(c)},$$

where *p*_*H*_ is the proportion of food that goes to a household, *H*(*c*) is the number of households in cell *c* and *p*_*W*_(*H*) is the proportion of food wasted at a household. We estimate the number of households in each cell *c*, *H*(*c*), using a Poisson distribution:

$$\begin{array}{l} H\left(c\right)\sim Pois\left(\overset{-}{H}\left(c\right)\right), \end{array}$$

where $\overset{-}{H}\left(c\right)$ is the expected number of households in cell *c*, determined by the number of people in cell *c*, *h*(*c*), divided by the average number of people per household in that country.

We now need to consider whether a backyard pig or boar could actually be in contact with this viral load. First we consider the situation for domestic pigs, by considering the viral load per household, *v*_*H*_(*c*), and the likelihood that a backyard pig would be in contact with it. To do this, we estimate the number of backyard pig farms (*N*_*BF*_(*c*)) in each cell *c*, using a Poisson distribution,

$$\begin{array}{l} N_{BF}\left(c\right)\sim Pois\left({\overset{-}{N}}_{BF}\left(c\right)\right). \end{array}$$

${\overset{-}{N}}_{BF}\left(c\right)$ is the expected number of backyard farms in cell *c*, which we outline how to compute in the Supplementary Info S1. The number of these backyard pig farms that swill feed their pigs is then estimated as

$$\begin{array}{l} N_{SF}\left(c\right)\sim Bin\left(N_{BF}\left(c\right),p_{SF}\right), \end{array}$$

where *p*_*SF*_ is the probability that a household with a backyard pig farm would illegally swill-feed their swine. This probability (see Supplementary Info S1) is not country-specific as sufficient data to be able to refine the parameter to country level are missing. *N*_*P*_(*c*) is the total number of backyard pigs in cell *c* which would swill feed, calculated as

$$\begin{array}{l} N_{P}\left(c\right)=\overset{N_{SF}\left(c\right)}{\underset{1}{\sum }}Pois\left({\overset{-}{N}}_{PF}\left(C\right)\right), \end{array}$$

where ${\overset{-}{N}}_{PF}\left(C\right)$ is the average number of pigs on a backyard farm which differs by country *C*.

Therefore, if a pig is swill fed, which is determined by the number of swill-feeding backyard farms *N*_*SF*_(*c*) and the number of pigs on those farms, *N*_*P*_(*c*), the amount of viral load that a single pig would have contact with is

$$\begin{array}{l} v_{P}\left(c\right)=v_{H}\left(c\right)\frac{N_{SF}\left(c\right)}{N_{P}\left(C\right)}. \end{array}$$

For wild boar contact with wasted pig meat products, there are two potential sources of wasted products that could end up in landfill or other sources of refuse—from a household that does not swill feed (whether it has a backyard pig farm or not) and from restaurants. We use the term landfill to denote any refuse site, including waste bins at household or in public locations. We define *v*_*L*_(*c*) as the viral load going to a landfill in cell *c* and compute it as

$$\begin{array}{l} v_{L}\left(c\right)=\;T_{W}\left(p_{L}\left(R\right)v_{R}\left(c\right)+\;p_{L}\left(H\right)\left(H\left(c\right)-N_{SF}\left(c\right)\right)v_{H}\left(c\right)\right). \end{array}$$

As *v*_*H*_(*c*) is the amount of viral load in product at a single household, we multiply it in the equation above by *H*(*c*)−*N*_*SF*_(*c*), the number of households in cell *c* that do not swill feed, to recreate the total amount wasted in cell *c* from those households. We also consider the proportion of waste that will be disposed of in a landfill or other rubbish disposal that wild boar can have contact with (compared to e.g., waste that goes toward biofuels), with separate proportions for restaurant waste *p*_*L*_(*R*) and household waste, *p*_*L*_(*H*). We also reduce the amount of viral load in a landfill site by the duration of time that the waste will be available for boar, *T*_*W*_, (as landfills are added to regularly, older waste will be difficult to access due to newer waste being placed on top; for other refuse sites, the waste will be taken away frequently). Due to lack of data on landfill locations, we assume that all waste is disposed of in the same cell as the waste was produced.

We now need to consider the likelihood that wild boar could have contact with this viral load at a landfill to estimate the amount of viral load each wild boar could be in contact with. To estimate this, we consider two probabilities—boar approaching and trying to contact landfill sites, *p*_*CW*_ and boar being able to gain access to the landfill sites, *p*_*AW*_. The number of boar trying to contact the waste site, *N*_*BW*_(*c*), is given by

$$\begin{array}{l} N_{BW}\left(c\right)\sim Bin\left(N_{TB}\left(c\right),p_{CW}\right), \end{array}$$

where *N*_*TB*_(*c*) is the total number of boar in cell *c*. The probability that contact will be successful depends on the access to the site, which is given by a Bernoulli distribution, leading to

$$\begin{array}{l} N_{B}\left(c\right)=N_{BW}\left(c\right)Bern\left(p_{AW}\right) \end{array}$$

as the number of boar accessing the site, *N*_*B*_(*c*), and eating food waste at a landfill. The accessibility to the waste will clearly be different depending on whether it is a landfill or a public bin near a forest, but we assume an average parameter across all types of refuse sites.

Lastly, we need to calculate the amount of viral load that a single wild boar could be in contact with in cell *c*, *v*_*B*_(*c*). We do this based on the approximation that an average boar will eat a household's weekly amount of waste (see Supplementary Info S1) and distribute the infected meat equally among households. This leads to

$$\begin{array}{l} v_{B}\left(c\right)=\frac{v_{L}\left(c\right)}{H\left(c\right)}. \end{array}$$

#### Transmission

For the viral transmission to pigs or wild boar, given contact with the contaminated meat, we assume an exponential dose-response relationship as this is both a common assumption in microbial risk assessment (42) and has been used to assess response to ASFV exposure (43). Given the viral load that a pig or boar could contact or ingest, we assume that a pig or boar will become infected given by the formula

$$\begin{array}{l} I_{p}\left(c\right)=1-e^{-rv_{P}\left(c\right)} \end{array}$$

shown here using the pig viral load, *v*_*P*_(*c*) as illustration, where *r* is the dose-response parameter from experimental studies.

#### Risk of Infection via Pig Meat Products

The equation above assesses if a single pig or boar will become infected due to the consumption of expected viral load that a pig or boar could be in contact with. We now combine this with the number of backyard pigs or boar at risk to calculate the probability of at least one infection in boar or pigs per year. Similar to the equation for *N*_*I*_(*c*) in the generic framework, we calculate *N*_*I,P*_(*c*), the number of infections in backyard pigs in each cell *c*, and *N*_*I,B*_(*c*), the number of infections in boar in each cell *c*, as

$$\begin{array}{l} N_{I,P}\left(c\right)=Bin\left(N_{P}\left(c\right),I_{p}\left(c\right)\right),\\ N_{I,B}\left(c\right)=Bin\left(N_{B}\left(c\right),\;I_{B}\left(c\right)\right). \end{array}$$

As indicated in the generic risk framework, the probability of at least one infection in boar, *R*_*I,F,b*_(*c*), or pigs, *R*_*I,F,p*_(*c*), in cell *c* via this pathway is estimated by the proportion of simulations in which *N*_*I,B*_(*c*) > 0 or *N*_*I,P*_(*c*) > 0, respectively.

### Overall Risk of Infection

The methods outlined above detail how the probability of at least one infection in boar or pigs is estimated per pathway. We combine these results, which are independently calculated, to produce the probability of at least one infection in boar or pigs in cell *c* via any of the three pathways considered. We define *R*_*I,P,s*_(*c*) as the probability of at least one infection in cell *c* via the trade in live pigs pathway in species *s* (either pigs or boar), *R*_*I,M,s*_(*c*) as the probability of at least one infection in cell *c* via the movement of wild boar pathway in species *s*, *R*_*I,F,s*_(*c*) as the probability of at least one infection in cell *c* via the trade in pig meat pathway in species *s*, and lastly *R*_*I,T,s*_(*c*) as the probability of at least one infection in cell *c* via any of the three pathways in species *s*. Then we calculate *R*_*I,T,s*_(*c*) as.

$$\begin{array}{l} R_{I,T,s}\left(c\right)=1-\left(1-R_{I,P,s}\left(c\right)\right)\left(1-R_{I,M,s}\left(c\right)\right)\left(1-R_{I,F,s}\left(c\right)\right). \end{array}$$

### Data

We include details of datasets used in the three pathways in Table 1. This accounts for the data that differed by country or by cell, whereas data that was represented by a single number or a distribution, primarily disease-related parameters, are provided in the Supplementary Info S1.

**Table 1:** The datasets used for three pathways, with a detailed description of the data used, the date the data is from and a reference for the data source.

**Model input**	**Data used**	**Date range**	**References**
Number of legal live pigs traded	TRACES data which states origin country, post-code location of end location, and number of pigs traded in a year.	2017	(35)
Prevalence of ASF in pigs	Country-level results from Simons et al. (33) which estimates prevalence using reported cases of the disease (from the OIE), under-reporting, the likelihood of an outbreak, the latent period of disease and stopping trade after disease is detected	Reported cases up to and including all of 2018	(33, 36, 44)
Prevalence of ASF in wild boar	Estimated prevalence at a 100 km^2^ cell level using reported cases of the disease and an under-reporting factor.	All wild boar cases in 2018	(44)
Wild boar abundance	A map of wild boar abundance and habitat suitability across Europe at a 1 km^2^ cell	2015	(38)
Amount of pig meat traded	Comext data from Eurostat which outlines country of origin, country of destination and amount in tons for many different product types related to pig meat.	2017	Comext data from Eurostat (40)
Number of pigs	Eurostat data on the number of pigs in each NUTS2 region	2017	agr_r_animal data from Eurostat (40)
Number of pig farms	Eurostat data on the number of pig farms in each NUTS2 region	2016	ef_lsk_main data from Eurostat (40)
Number of Backyard Pigs	Eurostat data on the average number of pigs on a backyard farm	2010	Pig farming sector—statistical portrait 2014 (40)
Number of Backyard Pig Farms	Eurostat data on the number of backyard pig farms in each EU MS	2010	Pig farming sector—statistical portrait 2014 (40)
World Map	Accurate world map indicating boundaries of countries	2017	Made with Natural Earth
NUTS map	Map of NUTS1 and NUTS2 regions in Europe	2018	(45)
Human density	A world map of the human population at a 1 km^2^ cell level	2015	(46)
Pig density	A world map of the pig density at a 5 km^2^ cell level	2014	(47, 48)
Number of households	Number of households in each EU MSs	2017	lfst_hhnhtych data from Eurostat (40)
Population	Population size in each EU MSs	2017	demo_pjan data from Eurostat (40)

*All the data is freely available apart from the TRACES dataset, in which access is via competent authorities within the TRACES network*.

### Sensitivity Analysis

We perform a sensitivity analysis of key parameters with the most uncertainty in the food pathway. In particular we analyze the effect of uncertainty on the following parameters: probability that food is cooked sufficiently to kill the virus (*p*_*C*<60_), the proportion of food that is wasted in a household or restaurant (*p*_*W*_(*H*), *p*_*W*_(*R*)), the probability of illegal swill-feeding (*p*_*SF*_), the accessibility of landfills for boar (*p*_*AW*_) and the duration of waste availability (*T*_*W*_). We measure the sensitivity of the food pathway to these parameters by focusing on those cells which are hotspots of disease risk, which we define to be any cell which has a probability of infection in boar of 0.02 or higher, or a probability of infection in pigs of 0.0001 or higher. For full details of the sensitivity analysis, see Supplementary Info S3.

## Results

The risk of infection with ASFV for pigs and wild boar is calculated for all EU MSs at a 100 km^2^ level for each pathway and also combined to produce an overall risk for each species.

### Overall Risk

**Figure 3:**
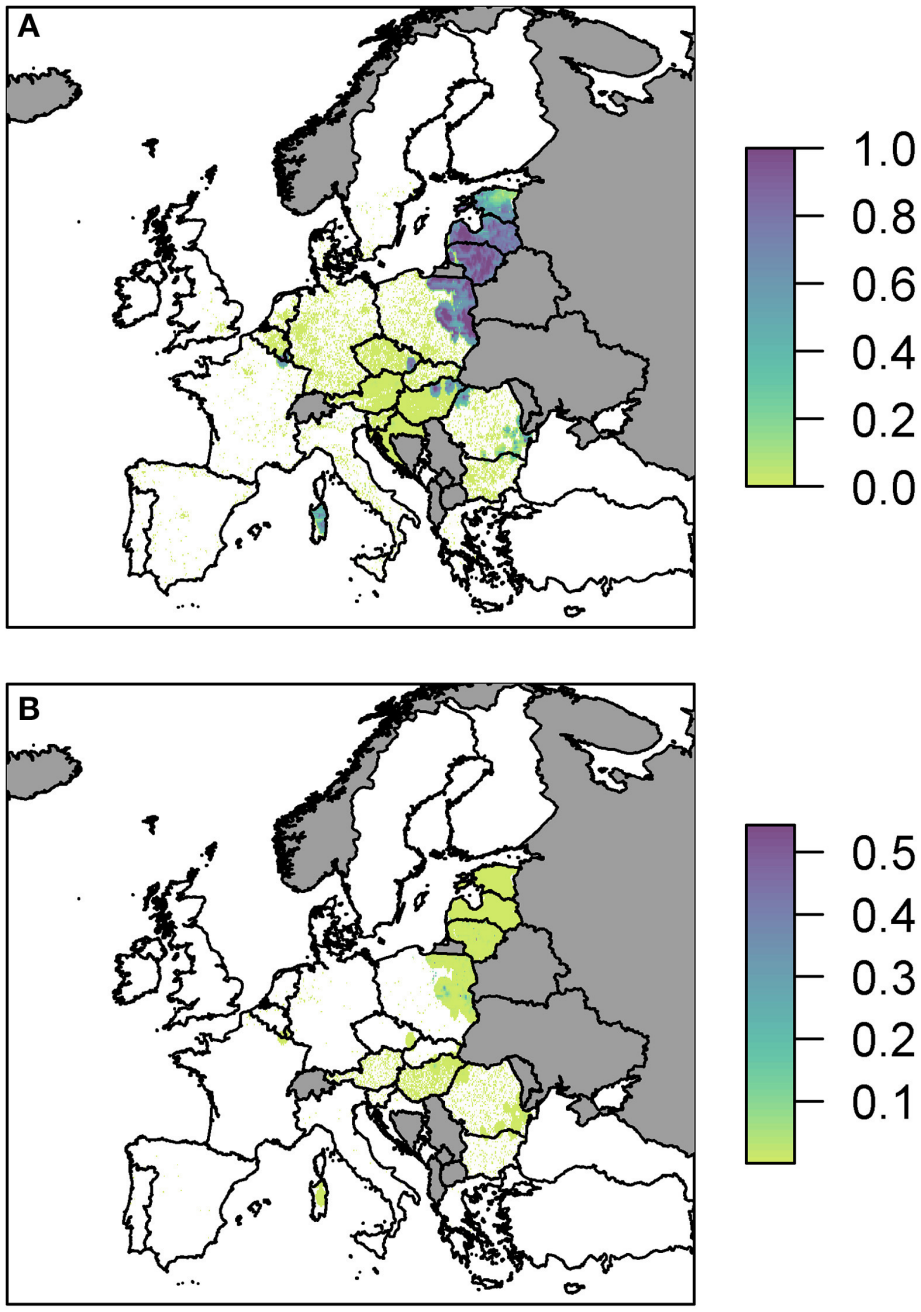
The probability of at least one infection with ASFV in **(A)** wild boar or **(B)** pigs in 2019, plotted at a 100 km^2^ cell level across Europe. Countries in gray have insufficient data to compute the risk.

We plot the overall probability of at least one infection in wild boar and pigs for all three pathways combined (Figure 3).

Predominantly, for both wild boar and pigs, the risk is focused in Eastern Europe with Latvia, Lithuania and parts of Poland in particular having many cells of high risk. There are also high risk areas in the east of Hungary and parts of Romania. Most countries have some areas of risk for wild boar transmission while transmission to pigs is much less frequent. This does not necessarily mean there is no risk for pigs in those countries, but the risk is too low to see on the scale forced by the high risk in other countries, combined with the fine spatial scale meaning it may be difficult to see the cells with very faint color. Considering the risk per pathway, below, allows us to investigate this in more detail. However, a detailed outline of how many cells fall within the different probability categories for the combined risk and for each pathway is provided in Supplementary Info S2. We also considered the risk when aggregated up to a country level, to compare the highest risk pathway for each country for infection in both wild boar and pigs (Supplementary Info S2).

### Legal Trade of Live Animals

**Figure 4:**
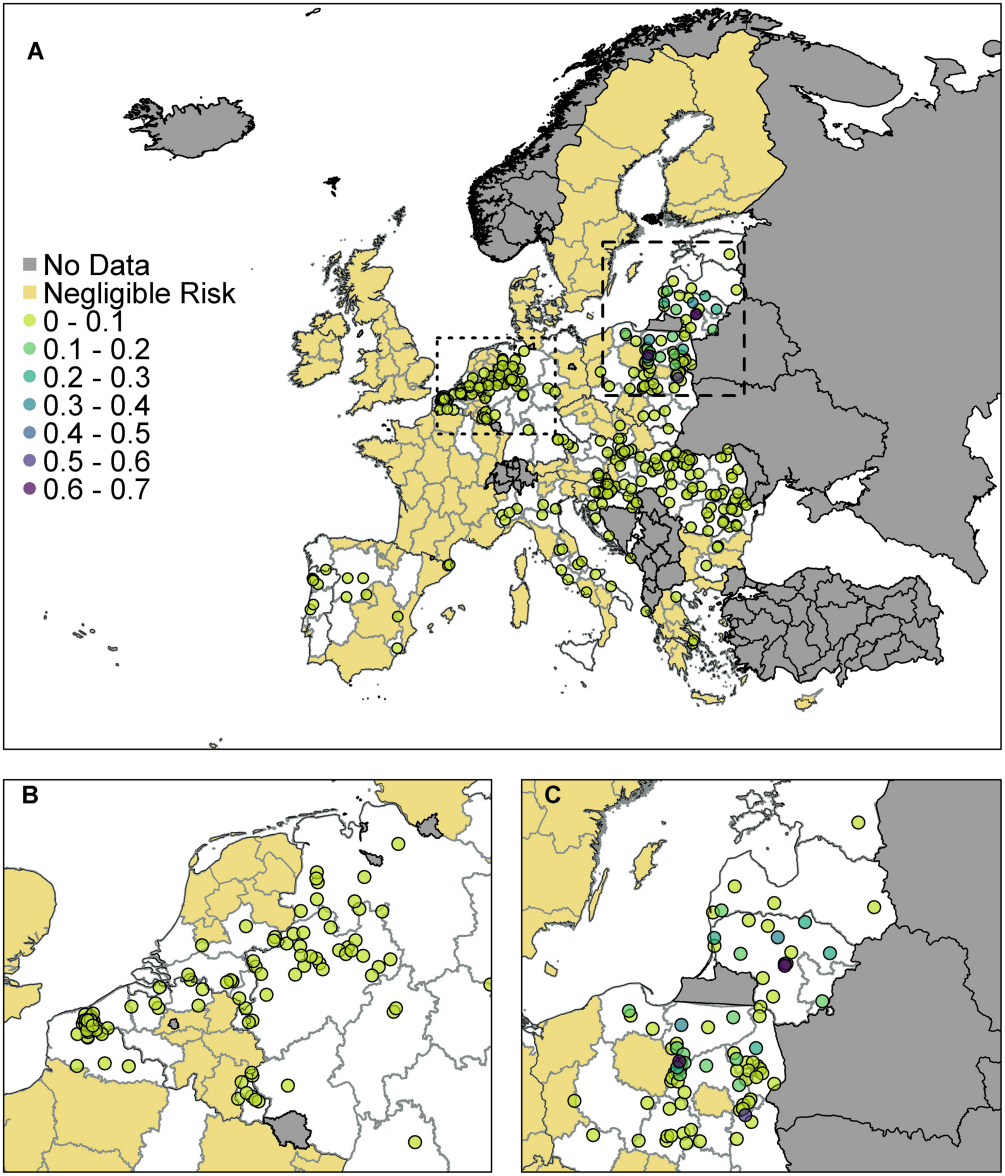
The probability of at least one infection of ASFV in pigs in 2019 from trade of live pigs at a farm level. In **(A)** all of Europe is plotted while in **(B)** the map is zoomed in to the dotted rectangle in **(A)** and in **(C)** the map is zoomed in to the dashed rectangle in **(A)**. All farms indicated by a circle imported at least one infected animal in at least one simulation and the color indicates the probability that one or more susceptible pigs became infected. Countries in gray have insufficient data to complete the risk assessment. All farms in the regions with negligible risk either did not import any pigs or did not import any infected pigs. ^©^EuroGeographics for the administrative boundaries.

The probability of at least one infection in pigs at a 100 km^2^ cell level for EU MSs is plotted in Figure 4. The original data for trade in live pigs was provided at a post-code level, and since there are only a few locations with a non-zero probability, in comparison to the size of Europe, we show these results using points to represent the post-codes, rather than plotting at the cell level.

According to the model, many farms across the EU are not expected to import any pigs at all. Of those that do import, the probability of importing an infected pig was low on most farms, based on prevalence data. There were only 310 farms that had a probability of 1 × 10^−4^ (i.e., one case expected in 10,000 years) or higher of importing an infected pig. We assume negligible risk in Figure 4 for any farm with probability <1 × 10^−4^ of importing an infected pig. Of those farms that do import, the probability of subsequent infection of a susceptible pig on most farms was low or very low; farms which have over a 10% chance of having at least one infection in a susceptible pig are in Poland, Latvia or Lithuania. The farm with the highest probability, at 65%, of at least one infection in a susceptible pig is in Lithuania and the pigs originated in Estonia.

As can be seen from Figure 4B, there are a large number of farms in the Netherlands, Germany, and France which have a small risk of infection occurring. In comparison (Figure 4C) fewer farms are at risk of importing infected animals in Poland, Latvia and Lithuania, but on those farms that do, the probability that this leads to infection is higher. This is primarily due to where the animals are imported from. Infected pigs imported by France, Germany and the Netherlands have primarily come from Belgium, which has a low estimated prevalence of infection in pigs for 2019 due to the wild boar cases in 2018. However, infected pigs entering Poland, Latvia and Lithuania are primarily coming from Latvia, Lithuania, and Estonia which have higher prevalence estimates.

When we combine all farms in a country together to compute a probability of at least one infection per country (Supplementary Info S2), we find that Poland has the highest risk with a 99.4% chance that at least one infection will occur on any farm in Poland from live pig trade. Lithuania also has a very high probability of 97.6%. There is then a drop to a 25% probability for Latvia, followed by Hungary, Germany, and the Netherlands, which has a 5% chance overall.

### Movement of Wild Boar

**Figure 5:**
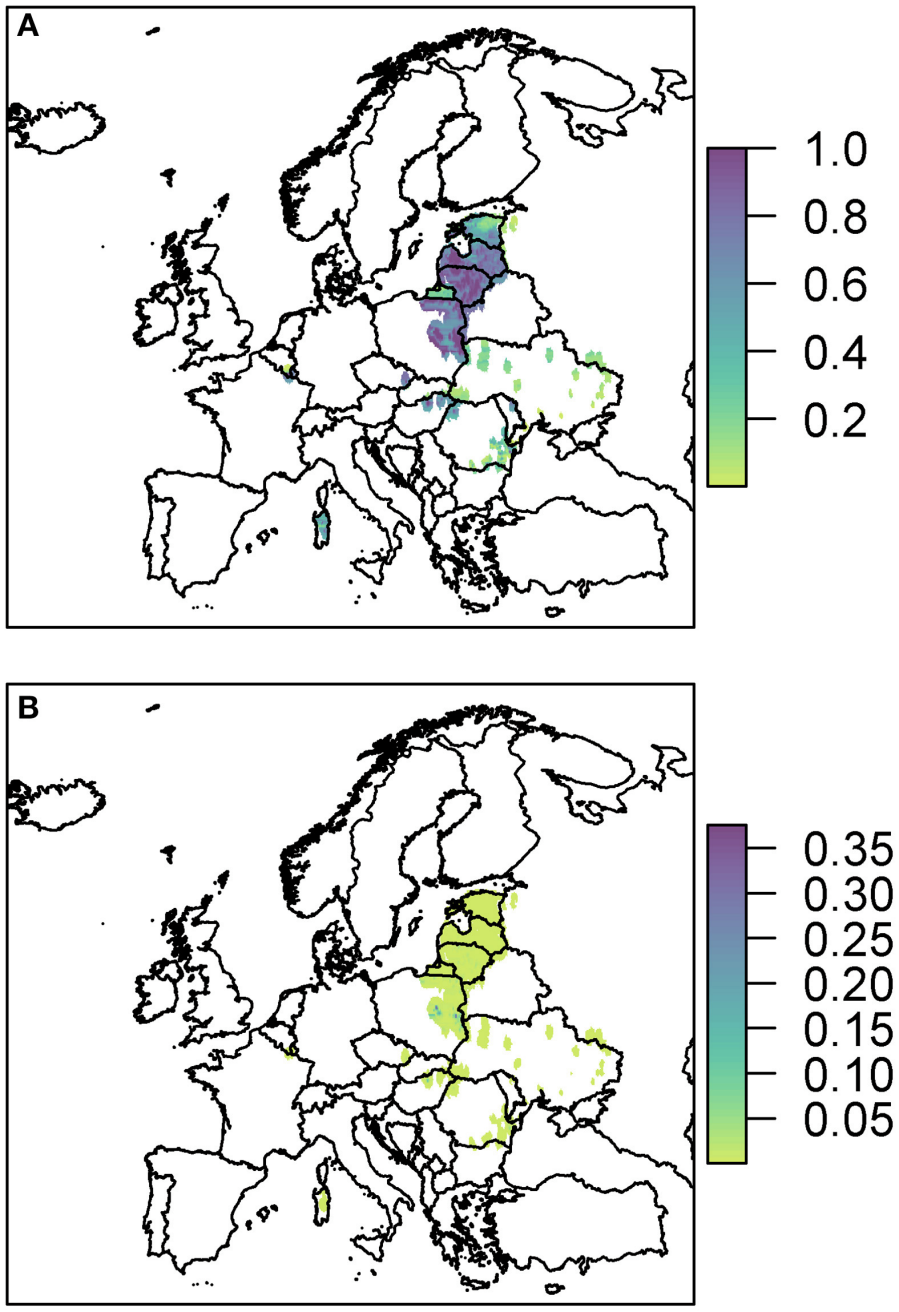
The probability of at least one infection of ASFV in 2019 in boar **(A)** and pigs **(B)** due to wild boar movement is plotted at a 100 km^2^ cell level across Europe. Prevalence in wild boar is estimated from reported cases in 2018.

The probability of at least one infection in wild boar or pigs via entry through the movement of wild boar is shown in Figure 5.

**Figure 6:**
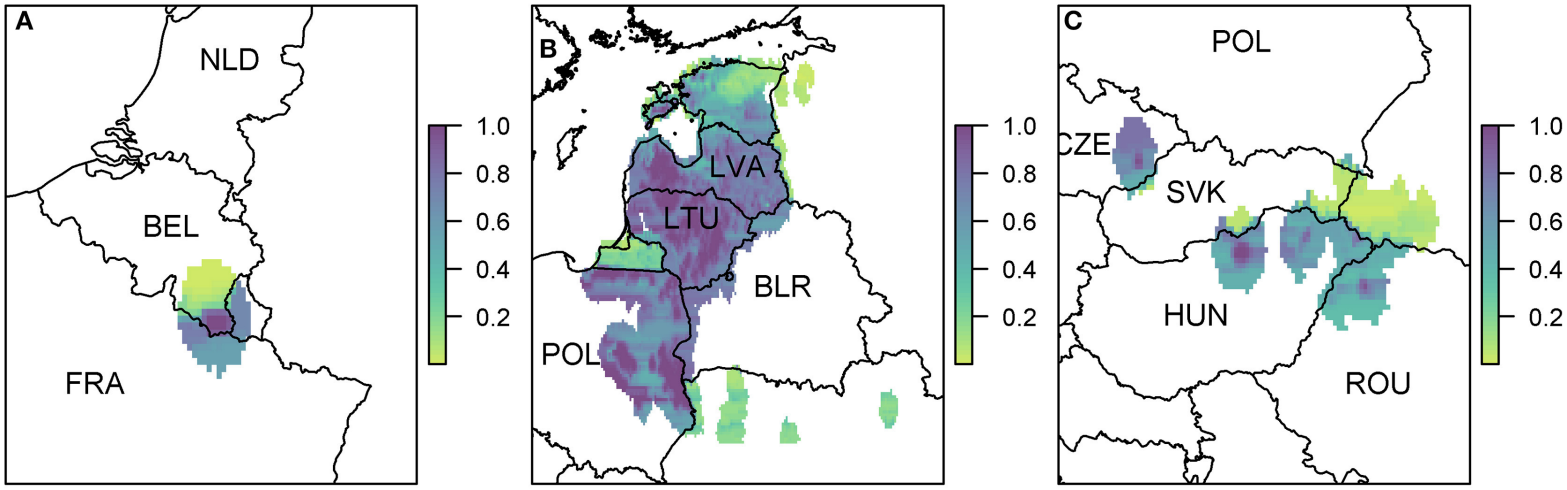
The probability of at least one infection of ASFV in boar in 2019 due to wild boar movement at a 100 km^2^ cell level. We zoom in to three regions where there were cases in 2018 **(A)** Belgium; **(B)** Poland, Lithuania, and Latvia; and **(C)** Hungary, Czech Republic, and Romania. Countries are indicated by their ISO3 code.

The risk to boar or pigs is very similar in terms of location, as all the risk is focused on the areas of reported cases in 2018. Wild boar movement is relatively local, with dispersal over long distances unlikely (49), and hence the disease is not expected to spread far due to boar dispersal (37). In order to see this more clearly, we zoom in to three regions to gain a better perspective of the risk to various regions in Europe due to wild boar movement (Figure 6). The distinct outbreak in Belgium demonstrates most clearly this risk region around previous cases, with a hotspot of high risk where the Belgium cases in 2018 were located, surrounded by an area of higher risk. However, this risk region is not symmetric as the boar movement is determined by habitat suitability, and infection of other boar depends on where boar are in the area—Figure 6A indicates that there is a higher risk to the south of the original cases toward France than north of the original cases.

Due to the high prevalence of cases in Poland, Latvia, and Lithuania in 2018, there is a large risk region throughout these countries (Figure 6B). As there were so many cases, the risk regions around each local outbreak are no longer distinct and hence there is a broader pattern of where infection could occur. We also highlight the area on the border of Hungary and Romania (Figure 6C) where there were cases in wild boar in 2018. There were many cases of ASF in Romania in 2018, but they were predominantly in pig farms, and hence our estimate for wild boar prevalence is relatively low, indicating not many cases of ASF in wild boar in Romania in 2019 due to wild boar movement.

The risk to domestic pigs due to wild boar movement is lower than the risk to wild boar (Figure 5). This is because the probability that wild boar will have contact with domestic pigs is very low as is the likelihood that this contact will lead to viral transmission—transmission via direct contact is most efficient via blood contact e.g., due to animals fighting, which is less likely to occur between boar and pigs compared to within boar groups. However, some Eastern European countries still end up with a very high probability of at least one infected domestic pig through this route; Poland has the highest risk with a predicted 100% chance of infection in pigs, followed by Lithuania (95%), Romania (80%), Hungary (77%), and Latvia (46%).

### Legal Trade of Pig Meat Products

**Figure 7:**
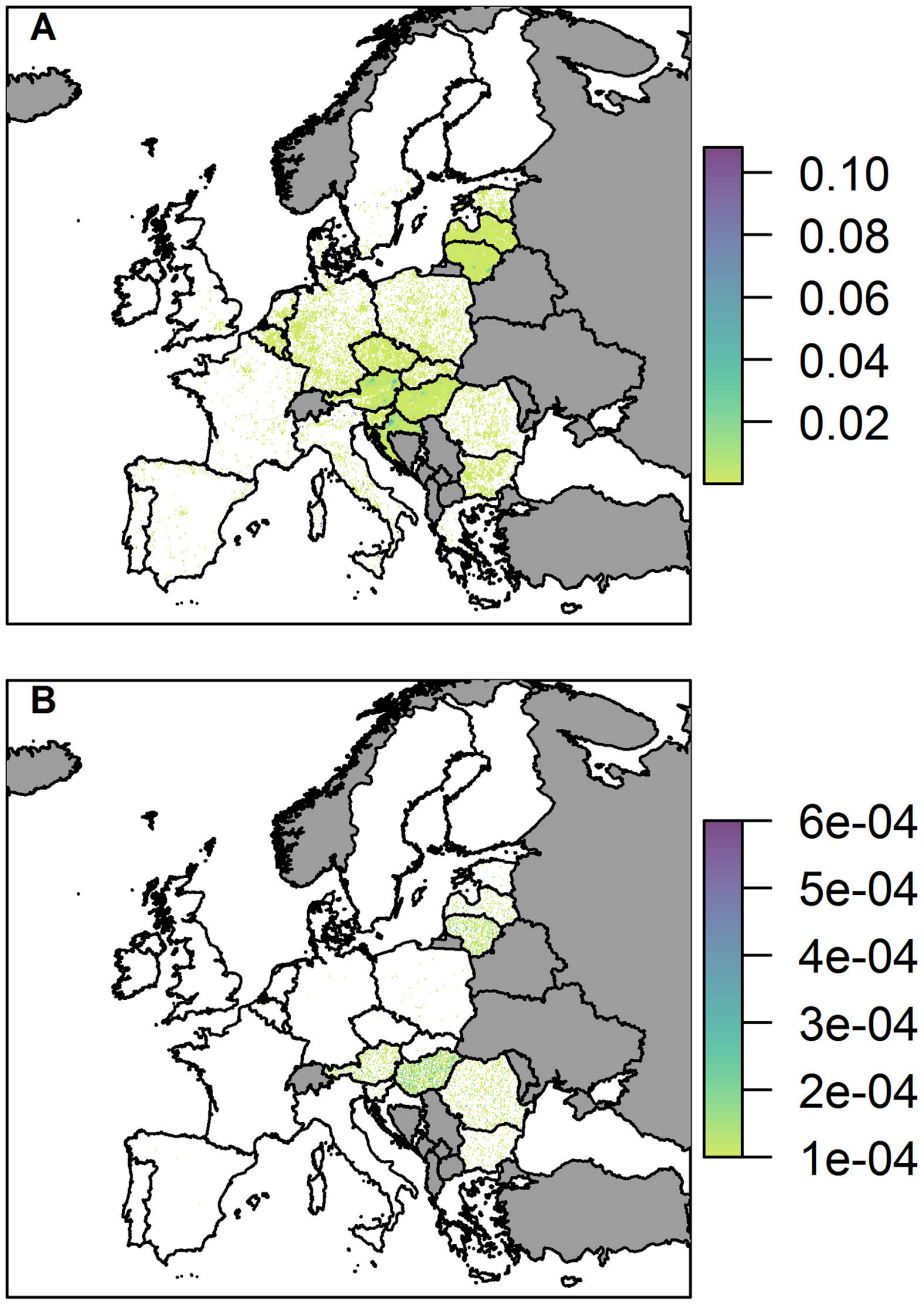
The probability of at least one infection of ASFV in 2019 in **(A)** wild boar and **(B)** pigs, via trade in legal pig meat products, plotted at a 100 km^2^ cell level across Europe. Countries in gray have insufficient data to complete the risk assessment.

We present the probability of at least one infection in wild boar and pigs via the legal trade in pig meat products pathway (Figure 7).

The probability of at least one infection in pigs via the trade in legal pig meat products is very low overall, with the highest risk in any cell being 6 × 10^−4^, noticeably lower than the highest risk via the two other pathways in a single cell. This risk is also focused mainly on a few countries; Hungary, Romania, Lithuania, Austria, and Latvia. For Lithuania, Austria and Latvia, this is primarily due to the amount of meat from infected pigs entering the country; for Hungary it is a combination of amount of trade and a high average number of pigs on each backyard farm; for Romania it is due to the considerably larger number of backyard farms in the country (more than 10 times any other country in Europe). At the country level, Hungary has the highest risk with a probability of 14.5% of at least one infection in pigs (Supplementary Info S2). The risk of ASF in boar via this pathway, in comparison, is much higher, and many countries across the whole of Europe have hotspots of higher risk (Figure 7B). This is because imported meat is more likely to end up at a landfill than being swill fed to backyard pigs. Although Hungary, Lithuania, Austria, Croatia, and Latvia have the most cells within their country at risk, giving the impression from the map that they may have the highest risk, the country with the highest risk for boar for this pathway is actually Italy. It has a probability of 99.7% of at least one infection in boar due to a small number of high risk cells, including in Sardinia.

### Pathway of Highest Risk

**Figure 8:**
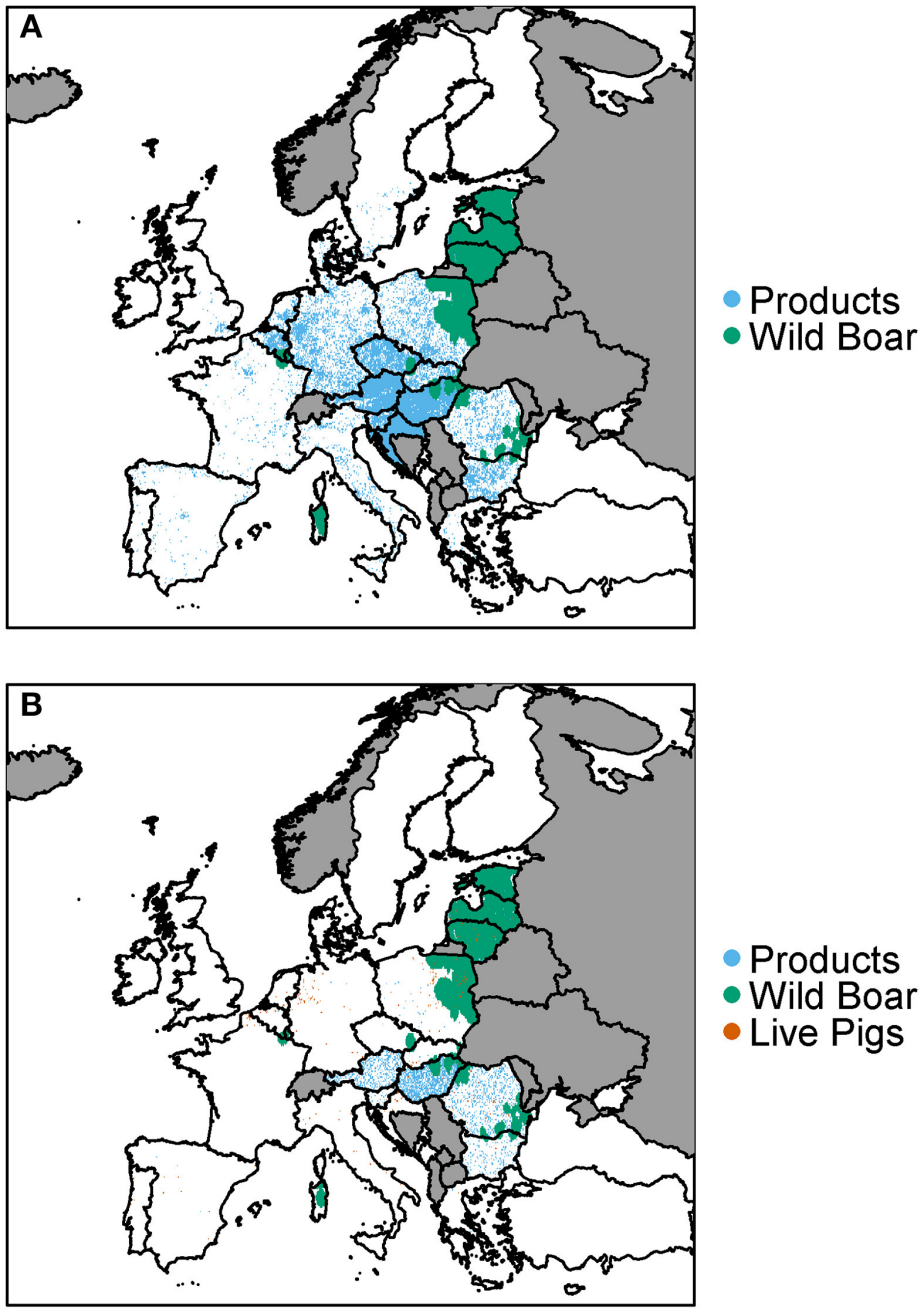
The pathway which has the highest risk of infection of ASFV for **(A)** wild boar and **(B)** pigs is plotted at a 100 km^2^ cell level across Europe. Countries in gray have insufficient data to complete the risk assessment.

For each 100 km^2^ cell we plot which pathway is the highest risk for that cell, for both wild boar and pigs (Figure 8), indicating at a finer scale where resources for different pathways should be focused. For most cells where wild boar movement can lead to infection in wild boar or pigs, this will be the pathway of highest risk. The legal trade of pig products pathway dominates as the highest risk pathway for infection in wild boar or pigs for many countries across Europe, with a focus on central Europe (e.g., Hungary, Austria, Germany, and Croatia). Dispersed throughout Europe are cells in which the legal trade of pigs is the highest pathway, highlighting the fact that this pathway is not widespread across Europe, due to the small number of farms importing infected pigs but, when it does occur, it is usually the riskiest pathway in that cell.

### Sensitivity Analysis

**Figure 9:**
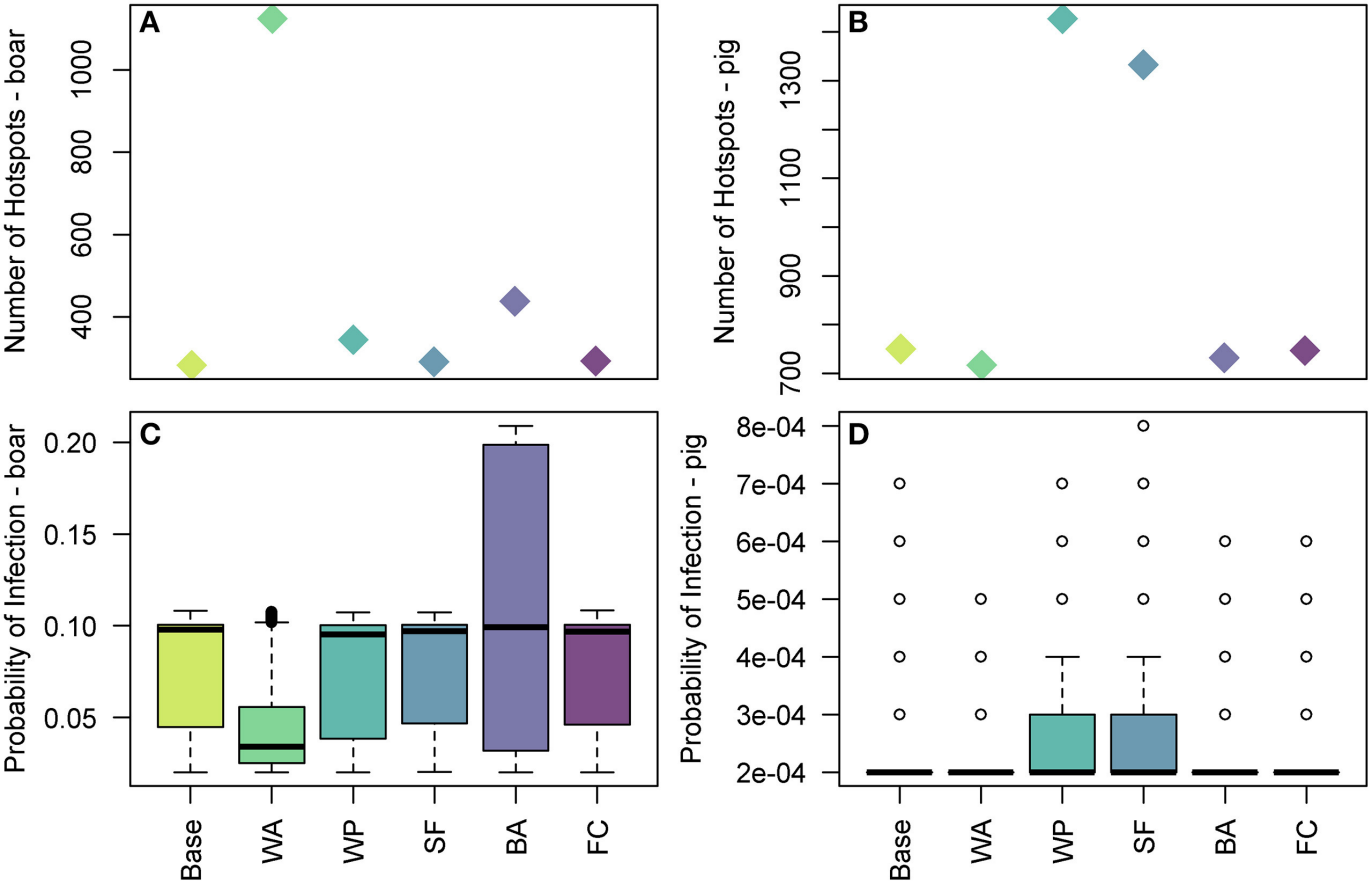
The effect of uncertain parameters in the legal trade of pig meat products pathway on hotspots of disease risk across Europe. In **(A,B)** the number of 100 km^2^ cells which are hotspots for wild boar infection and domestic pig infection are plotted, respectively, for the baseline and sensitivity parameters. In **(C,D)** the distribution of the probability of infection across 100 km^2^ cells which are hotspots in Europe for wild boar and pig infection, respectively, for the baseline and sensitivity parameters. The baseline results, as shown in Figure 7, are denoted by Base. The sensitivity parameters are: WA, duration of waste availability for boar; WP, proportion of food that goes to waste; SF, the probability of illegal swill-feeding; BA, the probability that boar are able to access waste; and FC, the proportion of food that is cooked sufficiently to kill the virus.

We measure the sensitivity of the food pathway to changes in 5 uncertain parameters in Figure 9 by considering changes to the hotspots of disease risk in the baseline results. For the wild boar, we find that the duration of waste availability (sensitivity WA) and boar accessibility to landfills (BA) are the two most influential parameters. BA increases the maximum of the probability of infection in boar, up to 0.2 in some cells, although the median is still similar to the baseline results. WA leads to a larger number of cells across Europe considered a hotspot, i.e., probability of infection > 0.02 (Figure 9A). However, although these new cells are hotspots, in general the probability of infection in these cells are in the lower range (i.e., most of these cells have a probability of infection around 0.05). As such, the median of the overall distribution of the probability of infection in the hotspot cells is lower (Figure 9C), although the highest risk cells (i.e., cells with probability >0.09) still exist. This is clearer to see in the map across Europe of the probability of infection in boar and pigs for the sensitivity analysis, provided in Supplementary Info S3.

For infection in pigs, the probability of illegal swill-feeding (SF) and the proportion of meat that goes to waste (WP) are the two most influential parameters as they both increase the number of cells considered hotspots i.e., probability of infection >0.0001 (Figure 9B) and increase the average and maximum probabilities of infection in those hotspots (Figure 9D). For both boar and pigs, the model is not sensitive to the probability that food is cooked sufficiently to kill the virus (FC).

## Discussion

ASF is a porcine disease of significant concern worldwide that is currently devastating the pig industry with reports that pig meat production has dropped by 30% in 2019 in China alone (50). The reduction in trade and subsequent costs are amplified by the cost of control, such as culling, cleansing, and disinfecting, implementation of fencing and increased hunting, and the costs of prevention, such as increased surveillance, dissemination of disease information and suggested/required prevention measures for farmers and the larger public (10). Therefore, prioritization of these measures is wise in order to utilize the available resources as optimally as possible. Risk assessments can be useful tools to provide a means for determining where the risk of disease is highest and which pathways are of most concern. They can indicate where the best places are to perform surveillance to prevent disease entry, or ensure quick and timely discovery of disease to allow the possibility of eradication before the disease infects the pig population at large in a country or becomes endemic in the wild boar population. In this study, we have performed a risk assessment for ASF in EU MSs that assesses the risk for 2019 at a fine spatial scale for three pathways in order that surveillance can be targeted to disease risk hotspots that are dependent on the pathway of entry. Overall, we found that the highest risk of infection in 2019 by ASFV via the three pathways of legal trade of pigs, movement of wild boar, and legal trade of pig meat products, is in Eastern European countries where the cases have so far been concentrated. This is driven by the wild boar movement pathway and the legal trade of live pigs. For the wild boar movement pathway, this is focused on Eastern Europe primarily due to the fact that boar movement is relatively local. For the legal trade of pigs pathway, analysis of the pig trade input data suggests that many European countries trade more with their neighbors, so Eastern European countries which border ASFV infected countries are more likely to trade with them and hence have a higher risk than countries geographically further away.

The risk of ASF in boar or pigs differed by spatial location and in magnitude. The risk of ASF in boar was higher than for pigs across the two pathways of relevance (trade in pig meat products and movement of wild boar) and as an overall risk, and was more broadly spread across the whole of Europe. While EU MSs main concern regarding ASF may be to protect their pig industry from incursion of ASF, given the ability of ASFV to transmit from wild boar to pigs once it enters a country, our results suggest that EU MSs should also focus on surveillance and prevention in wild boar populations as the disease is more likely to enter via this source. This is in line with observed disease incursions within Europe, as ASFV has been found in most EU countries in the wild boar population first [e.g., Poland, Hungary, Czech Republic, and Belgium (16)]. For countries without ASF in wild boar populations already, model results suggest the risk in boars is driven by the legal trade of pig meat products and hotspots occur where there is high boar density or high human density. However, there is high uncertainty associated with these absolute values due to uncertainty in the underlying data. The underlying wild boar abundance map (38) made good use of limited data but is known to have some issues, for example predicting no wild boar on the border of Finland and Russia and overestimating the abundance in close proximity to large cities in countries like the UK which are known to only have a few very specific localized boar populations. This is due to the need to extrapolate wild boar abundance across the whole of Europe with data collected only from some locations.

The results for the legal trade of pigs pathway indicate that 310 farms have a 1/10,000 risk of an infected pig entering the farm or higher. If this many farms were actually importing infected pigs each year, we would expect many more ASF cases on pig farms via import than has been seen over the past few years. The model may be overestimating this risk as we are not including an inspection, testing or quarantine step in the pathway, which would reduce the risk if it does occur. Secondly, rather than using the raw OIE data for the ASF prevalence in pigs in each country we use an algorithm designed to account for data gaps and underreporting (33). This algorithm predicts the prevalence of ASF in 2019 based on historical cases up to and including those in 2018, and may overestimate in some situations. These results indicate that Poland and Lithuania have lower numbers of farms importing infected pigs, but each farm imports more infected pigs and therefore have higher chances of infection on farm, compared to the Netherlands, France, and Germany, which have many farms importing but each farm has a low probability of importing an infected pig and it subsequently leading to transmission (Figure 4). Thus, this could lead to different risk-based prevention in trade for each area. Poland and Lithuania would reduce their risk by trading with countries with lower prevalence of disease, while Germany, the Netherlands, and France would reduce their risk by having fewer farms importing fewer pigs.

Results indicate that the spread of ASF via movement of wild boar is limited to short distances (37), although ASF is able to cross borders easily due to the lack of detection of infected wild animals. The risk via this pathway is much patchier, with many countries not affected at all in their pig or boar populations. The risk to wild boar is much higher than for pigs, due to the lack of contact between boar and pig populations. However, transmission to pigs can occur which can be reduced by ensuring good biosecurity practices are in place on pig farms to prevent the contact between wild boar and domestic pigs (2, 22). One aspect of this pathway which has not been included is any control measures that may have been implemented around reported cases of ASF in wild boar. For example, in Figure 6A, the risk of ASF in boar in France due to wild boar movement from the Belgian 2018 cases is relatively high. However, we did not include the fact that Belgium erected a fence around their reported cases to stop wild boar movement and implemented increased hunting in the area (16). Therefore, in reality, the risk to France would be much lower, depending on the width and permeability of the fence (37).

The legal trade of pig meat products pathway indicates highest risk for pigs in Eastern European countries which have high numbers of both backyard pig farms and average number of pigs on a backyard farm (40). However, these countries still have a low overall risk in comparison to other pathways. The low risk for this pathway is due to low probability of swill-feeding, as it is illegal, and the low chances that meat from infected pigs will enter a household with a backyard farm.

As there are so many component parts in the legal trade of pig meat products pathway, this pathway is subject to the most uncertainty. This uncertainty can be delineated into three major sources—using data from outside the EU, making assumptions regarding the spatial distribution of data, and simplifying the complicated food and waste industries. Firstly, the use of data from outside the EU. It was not possible to always find relevant data for EU countries and so it was necessary to use data that had been gathered from other countries as a proxy. In particular, we used an estimate for Australia for the probability that swill feeding would occur (51). Given that swill feeding in Australia is illegal, as it is in the EU, we felt this to be a reasonable approximation. Regarding the proportion of products consumed in a restaurant, the proportion of waste lost in the distribution chain and the proportion of waste that goes to landfills, we used a study from the USA as again we could find no data for EU countries (52). We also used a different US study regarding the probability that food is cooked to 60°C to kill the virus (53). It is possible that there are significant differences between the USA and EU countries regarding cooking and waste practices. We made a number of assumptions regarding the spatial distribution of data within the EU. This included assuming an (almost) homogeneous distribution of backyard pigs in all EU MSs, based on analysis of the spatial distribution in Great Britain (see Supplementary Info S1). In reality, this may not be the case for some countries in Europe, for example Romania, have a huge backyard pig sector with most households having a backyard pig farm or being part of a community farm, whereas in Great Britain there are relatively few backyard farms. Therefore, it is possible that an increase in number of farms would change the spatial distribution. Similarly, we assumed that the waste is kept in the same cell as it was produced. On the one hand, wild boar can access waste from intermediate waste sites (such as household or park rubbish bins) and so this assumption is suitable. However, potentially the waste is then conveyed to a small number of large collecting points. However, as we did not have data on the location of landfill sites, it was determined better to assume it stayed in the same cell. There was also a need to assume that all EU MSs acted in the same way, when in reality there could be many heterogeneities at a country level and even a finer scale. For example, the waste procedures may be different across each country, or even within each country, and the accessibility of landfills or other waste sites by boar may differ across Europe. Similarly the probability of swill-feeding is likely to be different across countries within the EU but due to a lack of data we assumed the same probability for all EU countries. Lastly, in order to parametrize this pathway given little data, we made simplifications regarding the food and waste industries. For example, we assumed that raw food would undergo a cooking process only in the destination country, when it is possible that salting, drying, or smoking processes were subsequently undergone after entry to the destination country. If meat underwent different processes after entry, this would likely change the viral load in the products upon consumption. There is also a lack of data regarding the effect of processes, such as salting, on the viral load in products, other than the decay due to the length of time for the process. Similarly, we did not separately consider the role of commercial bodies, such as food producers, when distributing the meat in each country. We assumed that the “restaurant” setting would cover all waste of meat that is not from a household setting. Although this restaurant option would be closer to food production than households in terms of amount of food wasted and the proportion of wasted food that goes to landfill, there may still be differences. For example, food producers may be more likely than restaurants to send their waste food to the biofuel industry or to other alternative places to landfills (52).

Given the numerous uncertainties with the legal trade of pig meat products pathway, we performed a sensitivity analysis on the most uncertain parameters within this pathway. This revealed that the model was most sensitive for estimation of infection in wild boar to parameters related to the waste procedures—the availability and accessibility of refuse waste to boar. Publication of waste procedures across multiple countries may reduce this uncertainty. For estimation of infection in pigs, the probability of illegal swill-feeding and proportion of pig meat products going to waste were the most sensitive parameters. It is very hard to reduce the uncertainty in the former parameter, due to its illegal nature, but data could be collated on the amount of food wastage across different food industry sectors to reduce the uncertainty in the latter parameter.

There are also uncertainty issues raised with the other pathways, for example, the map of wild boar abundance is based heavily on hunting bag records which are difficult to produce abundance estimates from accurately (54). Other uncertainties exist regarding boar ecology and movement dispersal. Data on wild animals and on abundance of species is difficult to collect and to ensure its accuracy, and therefore uncertainties will always remain when modeling a disease involving a wild species. Risk assessment is a prediction process and therefore, when asking what potentially could happen, there will always be both model and data uncertainty, lack of data or up-to-date data, and inaccurate data for wild and livestock species. For a model with such a broad scope, it is expected that there will be reasonable concerns about the specific absolute values. However, we believe we have found the best data possible for this risk assessment of ASF in Europe in 2019 and that our relative results regarding general trends, spatial hotspots, pathways of greatest risk, and comparisons between boar and pigs are robust.

Our risk assessment considered what we thought to be the most important routes for entry or spread of ASF in EU MSs. However, there are other potential routes that could lead to infection with ASF in boar or pigs, such as intra-country trade of pigs, illegal trade of pigs, or pig meat, non-commercial movement of pig meat (for example, travelers legally bringing meat across borders in Europe), and the transport of fomites (2, 9). Whilst our spatial framework is applicable to these pathways, they are difficult to parametrize in a quantitative setting across Europe due to lack of within-country data, free movement across Europe (leading to a lack of data regarding movement between countries), the extensive road and rail network, and the illegal nature of some of the activities. Simons et al. (33) considered an illegal pig products pathway, as well as the three considered here, for the entry of ASFV into EU MSs and found that the amount of meat from infected pigs entering each EU MSs was usually lower via illegal routes than legal routes, although there was more uncertainty associated with the illegal route. A few models have considered some of these routes in a semi-quantitative or qualitative manner indicating that these pathways could potentially be of greater risk than the three considered here. In a recent study that considers the risk of infection with ASFV in the Netherlands and Finland using multiple models (55), a semi-quantitative Finnish model (NORA) found that human transportation was the pathway of the greatest risk for Finland, and the qualitative Swedish model (SVARRA) found that the indirect pathway (including transport, human travel, feed, and bedding) was equal or higher risk than the three pathways in this risk assessment. Less recently, Mur et al. (31) considered the risk of introduction to European countries for multiple pathways using a semi-quantitative approach and found that the illegal trade and transport/fomites pathways were the riskiest for some countries (for illegal trade: France, Germany, Italy, and the UK; for transport: Belgium, Estonia, Lithuania, and Poland). However, this is based on a very different disease situation, as there were far fewer cases in wild boar in 2013 and hence the risk via the wild boar movement is expected to have increased since then given the current disease situation, and may therefore change the riskiest pathway per country if recalculated today. All risk assessments struggle to estimate the risk by pathways which are very stochastic in nature, usually due to human behavior. These behavioral aspects, such as whether farmers will implement good biosecurity, whether travelers will listen to warnings about not transporting meat products, or whether a driver will use a certain rest stop to eat their ham sandwich, are rare and unpredictable, making a risk assessment, especially on a fine spatial scale, unreliable. Perhaps connections with social science are required to understand these behavioral decisions and hence disease pathways more fully. However, it is generally considered that human-mediated transmission, for example, by non-commercial movement of meat products or fomites on transport, is one of the most important pathways for ASFV transmission (2). Therefore, risk assessments need to find a way to assess these pathways as accurately as possible.

Within this risk assessment we have taken a high-level approach, for example by modeling wild boar dispersal as a single event rather than considering the intricate population dynamics of wild boar. When considering exact management or control strategies to implement, a bespoke model including these intricate details may be required. Our high-level approach was taken in order to keep the risk assessment generic. Therefore, the movement of wild boar pathway can also be used for other wild animals that may transmit a different pathogen via either dispersal or home range movements, by only changing a few parameters. Similarly, the trade of live pigs and pig meat can be adapted for other species and diseases and, therefore, multiple diseases can be assessed using this overall framework. This can speed up risk assessment, especially for emerging diseases with little information to model intricately, and is useful for directing risk prioritization of diseases, pathways or locations. Furthermore, our risk assessment is easily updated with new data and able to be re-run quickly, allowing for changes in the risk profile across Europe to be monitored.

Our risk assessment for ASF indicates hotspots of high risk for disease incursion and infection, such as the border between Hungary and Romania for both the wild boar movement and legal trade of pig meat products pathways, or the area surrounding Belgium for the legal trade of pigs and pig meat. It also indicates which pathways should be the focus in different areas. These results can aid decision makers and risk managers to determine what type and intensity of surveillance, prevention and control measures are necessary for different regions for ASF. This risk assessment will assist EU MSs in their efforts for the prevention and detection of ASF, and our risk assessment framework is applicable for other locations, such as China, Vietnam and Cambodia, provided the equivalent data are available. As ASF continues to spread throughout Europe and across Asia, risk assessments such as this can determine how to best tackle the disease.

## Data Availability Statement

All datasets generated for this study are included in the article/Supplementary Material.

## Author Contributions

RT, PG, LK, and ES developed the generic framework. RT developed the trade of live animals pathway. RT and RS developed the wild boar movement pathway. RT and RC developed the trade of products pathway. RT and RC wrote the manuscript with input from all co-authors.

### Conflict of Interest

The authors declare that the research was conducted in the absence of any commercial or financial relationships that could be construed as a potential conflict of interest.
