# Supplementary material for: The Risk of Infection by African Swine Fever Virus in European Swine Through Boar Movement and Legal Trade of Pigs and Pig Meat
Source: Front Vet Sci. 2020 Jan 9;6:486. doi: 10.3389/fvets.2019.00486 (PMC6962172; doi:10.3389/fvets.2019.00486)
Supplement: Supplementary file 1 [file Data_Sheet_1.pdf]

The risk of infection by African swine fever virus in European swine through boar movement and legal trade of pigs and pig meat
Supplementary Information

Rachel A. Taylor[1], Roberto Condoleo[1,2], Robin R.L. Simons[1], Paul Gale[1], Louise A. Kelly[1,3], and Emma L. Snary[1]

[1]Department of Epidemiology Sciences, Animal and Plant Health Agency (APHA), UK

[2]Istituto Zooprofilattico Sperimentale Lazio e Toscana "M. Aleandri", Italy

[3]Department of Mathematics and Statistics, University of Strathclyde, UK

Supplementary Info S1: Additional Methods

Further details on the methods and data used to calculate this risk assessment are provided here in order to aid the reader to understand the methods and be able to replicate our results if desired.

Legal trade of live pigs

The parameter values that are required for the legal trade of live pigs pathway, summarised in Table 1, are those that are involved in the equation for R_0. We estimate the contact rate between pigs on a farm based on a study by Temple et al. (2011) in which the behaviour of Iberian pigs was assessed on intensive and extensive pig farms. The percentage of animals engaging in social contact on extensive farms was 3.3% (±0.73) and on intensive farms was 15.1% (±1.51). Since pig farms across Europe may be extensive or intensive and we do not have data on which type the farm would be, we assume an average of 9.2% (±1.19). To convert this percentage into a yearly contact rate we multiply by 365.

The probability of transmission of ASFV from pig to pig given contact is estimated from experimental studies. From an experimental study which considered innoculation through a low dose of ASF such as through touching rather than blood contact (Pietschmann et al. 2015), we estimate 0.167 as the mode of a pert distribution. Since we expect that some contact may not be suitable for transmission, we set 0 as the minimum of the pert distribution. We assume a maximum of the pert distribution of 0.3 based upon studies by Guinat et al. (2016) and Pietschmann et al. (2015) in which successful transmission to pigs in direct and indirect contact can take many days to occur, despite the close contact involved in experimental settings. Therefore, it is reasonable to assume that not all contacts will be successful.

The length of the infectious period in pigs is taken as a pert distribution, estimated from experimental studies on ASFV infection in pigs (Gabriel et al. 2011, Guinat et al. 2014).

Lastly, we need to estimate the number of susceptible pigs that infected pigs will be in contact with. This is usually determined by pen sizes, which are highly variable within and between EU MSs. The study by Temple et al. (2011) indicated that pen sizes in intensive farms ranged from 7 pigs to 320 pigs while in extensive farms the average size was 185 pigs. We also have data on the number of pigs in each region and the number of farms through which we can calculate the average number of pigs on a farm in each region. We therefore assume a uniform distribution for the number of susceptible animals that ranges from 7 up to the minimum between our calculated average farm size and 320 pigs.

Table 1 Parameter values for the legal trade of live pigs pathway with a description and reference. Rates and times are given in units of years unless otherwise specified.

Parameter	Value	Reference
Contact rate between pigs on a farm	Norm(0.092, 0.0119)*365	Temple et al. (2011)
Probability of transmission between pigs given contact	Pert(0, 0.167, 0.3)	Estimated from Pietschmann et al. (2015), Guinat et al. (2016)
The length of the infectious period in pigs	Pert(3/365, 6/365, 10/365)	Based on Gabriel et al. (2011), Guinat et al. (2014)
Number of susceptible pigs	Unif(7, min(320, Average farm size in region))	Temple et al. (2011), Eurostat (2017)

Movement of wild boar

We estimate the incidence of ASFV infection in wild boar in each cell c using the reported cases of ASF in wild boar in 2018, an under-reporting factor and a smoothing method to estimate potential unreported cases in neighbouring cells. For exact details see Taylor et al. (2019b). The estimated incidence in Europe in 2018 is plotted in Figure 1.

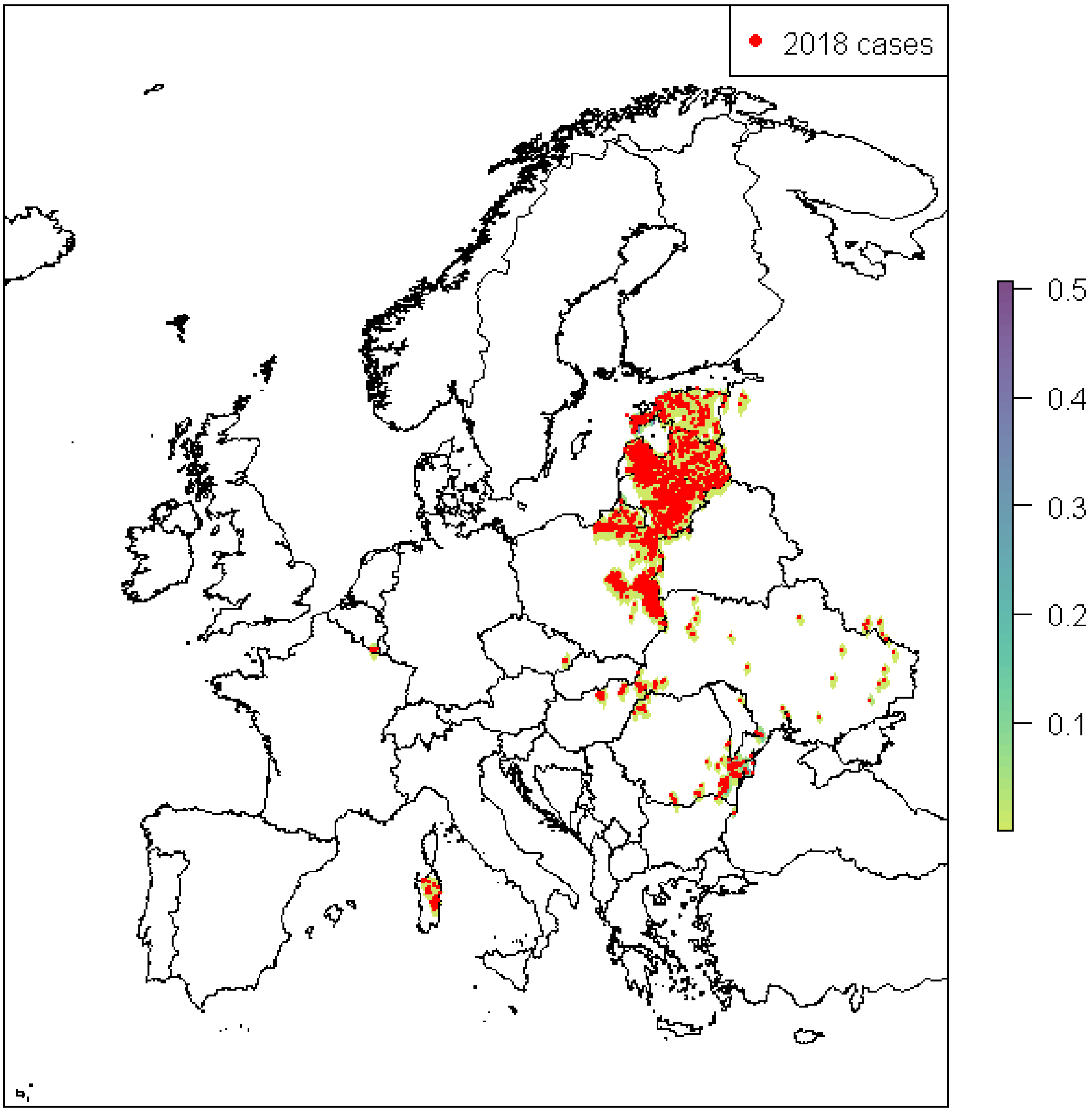

Figure 1 The estimated incidence of ASF in wild boar in 2018 in Europe at the 100km[2] cell level indicated by the colour scale. The reported cases in wild boar are plotted in red. The highest areas of incidence are in those areas with reported cases (hence are hidden behind the red circles).

As stated in the main text, there are multiple equations used for R_0 in the wild boar movement pathway depending on whether the wild boar are in contact with and transmit the infection to pigs or other boar. In line with Taylor et al. (2019b), we model the contact between live boar and susceptible pigs in each cell c by considering the contact rate (γ), the probability of transmission given contact (β), the number of susceptible pigs in the cell ($S(c)$) and the length of the infectious period in boar ($1/r$):

$$R_0(c) = \frac{\beta\gamma p_S S(c)}{r}.$$

Here, p_S is the proportional size of the boar home range compared to the cell size.

For wild boar contact with other boar, we consider the fact that wild boar normally exist in matrilineal groups (Podgórski et al. 2014), and so we adapt the contact rate to include both a within-group contact and a between-group contact. To do this, we replace $\gamma p_S S(c)$ with

$$\gamma_W G + \gamma_B (p_S S(c) - G).$$

Here, γ_W is the within-group contact rate, G is the average group size and γ_B is the between-group contact rate which is applicable for contact with all other boar in the home range.

A second consideration for wild boar transmission is the probability that wild boar will die from infection. The carcasses of ASF-infected animals can still be infectious and contribute to transmission of ASF. We assume that domestic pigs would not have any contact with boar carcasses, and thus this is only relevant to the R_0 equation for wild boar. We estimate the number of new cases of ASF due to contact with an infected carcass as

$$\frac{p_d \gamma_d \beta_c S(c)}{r_c}.$$

In this equation, p_d is the probability that direct contact will occur with a carcass, γ_d is the total number of direct contacts per year each boar has with a carcass, β_c is the transmission probability from a carcass to a susceptible animal per contact, r_c is the rate at which the carcass is available to cause infection, which is the inverse of the length of time the carcass is available (T_c). T_c is determined by two factors, skeletonisation of the carcass and whether the carcass is found and removed, as follows:

$$r_c = \frac{1}{T_c} = \frac{1}{(1 - p_r)T_S + p_r T_r},$$

where T_S is the time until skeletonisation of the carcass, T_r is the time until removal of a carcass and p_r is the probability that a boar carcass is found and removed.

Therefore, our full equation for $R_0(c)$ in susceptible boar populations, i.e. the likelihood of new cases occurring in susceptible boar in cell c given an infected wild boar has entered the cell, is:

$$R_0(c) = \frac{\beta(\gamma_W G + \gamma_B (p_S S(c) - G))}{r} + p_L \left(\frac{p_d \gamma_d \beta_c}{r_c}\right) S(c),$$

where p_L is the probability of lethal infection in boar.

Parameter values for the wild boar movement model and the transmission of the disease are in Table 2. For a description of why specific parameter values were chosen, please see Taylor et al. (2019b).

Table 2 Parameter values for the wild boar pathway with a description and reference. Rates and times are given in units of years unless specified otherwise.

Parameter Description	Value	Reference
Proportion of cell explored during home range movement (p_S)	0.05	Based on Massei et al. (1997), Leaper et al. (1999), Podgórski et al. (2014)
Number of steps during long range movement (n)	5	Truvé and Lemel (2003)
Proportion of boar performing long range movement	15.4%	Keuling et al. (2010)
Infectious period in live boar ($1/r$)	Pert(min=3/365, mode = 6/365,max=10/365)	Based on Gabriel et al. (2011), Guinat et al. (2014)

Probability of being infectious during long range movement (p_i)	8/365	Guinat et al. (2014)
Group size of wild boar (G)	7	Podgórski et al. (2014)
Per capita contact rate between boar and pigs in 100km^2 cell with live boar (γ_W, γ_B, γ)	Within group γ_W,: norm(0.59, 0.02)*365<br><br>Between group γ_B: norm(0.035, 0.002)*365<br><br>Boar to pig γ: unif(0, 0.267)	Podgórski et al. (2018)
Probability of transmission (β)	Boar to pig: unif(0,0.167)<br><br>Boar to boar: pert(0, 0.167, 0.3)<br><br>Dead boar to boar: unif(0, 0.167)	Estimated from Pietschmann et al. (2015)
Probability that ASF will be fatal in boar (p_L)	unif(0.95, 1)	Blome et al. (2012), Thulke and Lange (2017)
Probability that a dead boar will have direct contact with live boar (p_d)	0.5	Probst et al. (2017)
Yearly direct contact rate with boar carcass (γ_d)	1/370 *norm(179.5, 73)	Probst et al. (2017)
Length of infectious period for boar carcasses (T_s)	pert(15/365, 26/365, 124/365)	Estimated from Morley (1993), Probst et al. (2017), Olesen et al. (2018), Chenais et al. (2019)
Probability that a carcass will be removed	1/4	Estimated from the under reporting factor
Under reporting factor	4	Adkin et al. (2004)

Legal trade of pig meat products

Product Types and Composition

As outlined in the main text, there are many different pig meat product types that are traded to and from EU MSs. In total, there are 119 different product codes in Comext that correspond to some form of pig meat, not including those products which are not for consumption purposes or we considered as no risk, such as hides, dog or cat food, or canned meat (as we assumed it would be heated sufficiently to kill off any virus). Following Adkin et al. (2004) we simplify these into 12 categories, as seen in Table 3. There are 5 processes that could be applied to each of the product categories (dried, smoked, salted, chilled and frozen), but not all product categories undergo all processes. This leads to a total of 21 different product categories by type and process (Table 3).

Table 3 The simplified product types in 12 categories, the processes each product undergoes and the associated Comext products codes that we included in each category, based upon Adkin et al. (2004).

Product Type	Process	Comext Code
Dried de-boned meat	Dried; Smoked	2101981
	Salted; Dried; Smoked	21019
Dried meat bone-in	Dried; Smoked	21201131, 2101981, 21019, 2101219, 2101960, 2101970, 2101989
Fat	Chilled	209, 20900, 20900, 2090030, 20910, 2091011, 2091019, 2091090
	Dried; Smoked	2090019
	Salted	2090011
Frozen meat (bone-in)	Frozen	20321, 2032110, 2032190, 20322, 2032211, 2032219, 2032290, 20329, 2032913, 2032959, 2032990
Frozen meat (de-boned)	Frozen	2032911, 2032915, 2032955
Offal	Chilled	20630, 2063000, 2063010, 2063020, 2063021, 2063030, 2063031, 2063080, 2063090, 16010010, 1602, 160210, 16021000, 160220, 16022090, 160249, 16024919, 16024930, 16024950, 16024990, 16029051
	Chilled; Frozen	5100010
	Chilled; Frozen; Salted; Dried; Smoked	504, 50400, 5040000
	Frozen	20641, 2064100, 2064110, 2064120, 2064180, 2064191, 2064199, 20649, 2064900, 2064910, 2064920, 2064980, 2064991, 2064999
	Salted; Dried; Smoked	2109031, 2109039, 2109080, 2109090, 2109941, 2109949
Raw de-boned meat	Chilled	2031955
Raw ground meat	Chilled	160100, 16010091, 16010099
Raw meat bone-in	Chilled	16010099, 20311, 2031110, 2031190, 20312, 2031211, 2031219, 2031290, 20319, 2031911, 2031913, 2031915, 2031959, 2031990

Salted de-boned meat	Salted	2101951
Salted meat bone-in	Chilled	160241, 16024110, 16024190, 160242, 16024210, 16024290, 16024911, 16024913, 16024915
	Salted	2101111, 2101119, 2101211, 2101910, 2101920, 2101930, 2101940, 2101950, 2101959
	Salted; Dried; Smoked	21011, 2101190, 21012, 2101290, 2101990
Skin	Chilled	41033000

Each of these 12 product categories are composed of tissues in different proportions. These tissues include muscle, skin, offal, bone and fat. In Table 4 we outline the proportion of each product that we assume is a certain tissue, also based upon Adkin et al. (2004).

Table 4 The composition of each of the product types we consider. The proportion of each product type that is composed of muscle, fat, bone, skin and offal is indicated.

	Composition				
**Product Type**	**Muscle**	**Fat**	**Bone**	**Skin**	**Offal**
Dried de-boned meat	0.75	0.25	0	0	0
Dried meat bone-in	0.55	0.225	0.225	0	0
Fat	0	1	0	0	0
Frozen meat (bone-in)	0.55	0.225	0.225	0	0
Frozen meat (de-boned)	0.75	0.25	0	0	0
Offal	0	0	0	0	1
Raw de-boned meat	0.75	0.25	0	0	0
Raw ground meat	0.75	0.25	0	0	0
Raw meat bone-in	0.55	0.225	0.225	0	0
Salted de-boned meat	0.75	0.25	0	0	0
Salted meat bone-in	0.55	0.225	0.225	0	0
Skin	0	0	0	1	0

Transport Time

We estimated the transport time of trade of pig meat products from each origin country to each destination country based on a method from Simons et al. (2016). We did this in order to estimate the amount of time from slaughter to consumption and hence to determine the remaining viral load due to decay over time. The distance of an origin country to a destination country was calculated using the great-circle distance metric, which takes into account the curvature of the earth. We estimated the average speed of ”fast” transport such as by air, and ”slow” transport such as rail, road and ship, in order to calculate the time taken between countries via fast or slow transport. We then estimated the proportion of travel that occurred via fast or slow transport by analysing data on trade for each EU MSs from Eurostat (Eurostat 2017) to calculate an average time taken from one country to another regardless of transport method.

Backyard Pigs

An estimate for the number of backyard pig farms in each cell c is required, but there are no data on the exact locations of backyard pig farms in each EU MSs. However, EU MSs provide Eurostat with estimates of the total

number of backyard pigs and backyard pig farms in their country stratified by pig gender, size and age. We considered all backyard pig farms that did not have a sow to estimate the total number of backyard pig farms in each country and the average number of pigs on each farm.

We then need to estimate where the backyard pig farms are located. We had additional data for Great Britain regarding locations of backyard pig farms. We plotted this to discover if there were patterns or proxy data that we could use to estimate where backyard pigs are located. In general we found that the backyard pig farms were relatively spatially homogenous. We also found no correlation between backyard pig farm density and pig density. This makes sense as there is no reason to believe that backyard pig farms will be located near to commercial farms. We found a small correlation between human density and backyard pig farm density. In particular, we found that when human density is very low, there are unlikely to be backyard pig farms. Therefore, we analyzed the data for an effective cutoff which determined if human population numbers are high enough to host backyard pig farms. Otherwise, we assume a homogenous distribution of backyard pig farms. Thus, for all cells in each country that are above that human density cutoff, we distribute the number of backyard pig farms in that country evenly and assume no backyard pig farms in the cells below the cutoff. We assumed that all countries are similar to Great Britain regarding distribution of backyard pigs. This gives an estimate for the number of backyard pig farms in each cell c which we use within a Poisson equation to gain a simulated value for each cell.

Boar Dietary Habits

When calculating the amount of viral load that a boar could contact or ingest at a waste site, we are required to consider how much boar eat in comparison to how much food is present. According to Badminton Feeds (2019), wild boar eat up to 4kg of food per day, and the average UK household sends 4.6kg of food to landfill each week (WRAP 2008). We therefore assume that if a boar is able to enter a landfill/waste site, they will consume one household's amount of food at a time. We distribute the infected meat equally among households to estimate the total viral load in each household's waste and hence the amount each boar would eat at a time.

Parameter Values

The parameter values for the legal trade of pig meat products pathway are outlined in Table 5.

Table 5 Parameter values for the legal trade of pig meat products pathway with a description and reference. Rates and times are given in units of years unless otherwise specified.

Parameter	Description	Value	Data sources
Incubation period	Mean numbers of days before an infected pig manifests clinical signs.	10	Gulenkin et al. (2011), Blome et al. (2013), Sànchez-Vizcaíno et al. (2015), Arias et al. (2018)
Viremia peak day	The day when a viremia peak is detected in an infected pig.	5	Mebus et al. (1997)
Period of viral detection (day)	The day when the virus is not detectable anymore in an infected pig.	230	Hartnett et al. (2014)
Initial viral load after slaughter in each product type z:	Average viral load of several tissues from an infected pig	Muscle: 4.76<br>Fat: 3.89<br>Bone: 6.85	Mebus et al. (1997), Hartnett et al. (2014)

$v_I(z_p)$	during the incubation period in Log_{10} HAD_{50}/g	Skin: 3.89<br>Offal: 5.63	
Maximum virus survival capacity in food in days – $1/r_v(z)$	Maximum time that the virus could survive in products that have undergone different processes.	Chilling: 110<br>Freezing: 1000<br>Drying: 300<br>Salting: 182<br>Smoking: 30	Kovalenko (1964), McKercher et al. (1978), Blackwell (1984), McKercher (1987), Kleiboeker (2002), Hartnett et al. (2014)
Time of processing (t_p)	Time taken to undergo the food process (in days)	Chilling: 0<br>Freezing: 0<br>Drying: Pert(0.25; 7; 270)<br>Salting: Pert(0.25; 28; 270)<br>Smoking: Uniform(0.33;1)	Hartnett et al. (2014)
Fast transport proportion	Proportion of transport of food products between countries that occurs via fast transport methods e.g. air.	Normal(0.00325,0.006636)	Eurostat (2017)
Food is not cooked to at least 60°C ($p_{C<60}$)	Probability that the food is not cooked to at least 60°C, $p_{C<60}$.	0.2	EcoSure (2008)
Food lost along the food chain ($p_{L<\{H,R\}}$)	Proportion of food that is lost along the food chain prior to reaching a restaurant or household (during the processing, packaging and distribution phases)	0.09	FAO (2011)
Pig meat goes to a restaurant, (p_R)	Proportion of pig meat that goes to a restaurant	Normal(0.20, 0.03)	Herrera-Ibatá et al. (2017)
Food wasted at a restaurant ($p_W(R)$) or household ($p_W(H)$)	Proportion of food that is wasted at a restaurant or a household. Assumed the same values for household and restaurant	Normal(μ, 6.6)<br>Where μ is:<br>UK: 0.23<br>Netherlands: 0.08<br>Denmark: 0.07<br>Germany: 0.13	Vanham et al. (2015)

		Other EU MSs: 0.145	
Human density cut-off	The number of humans in a cell below which backyard pig farms are not expected	200	Estimated using data from Great Britain
Illegal swill-feed (p_{SF})	Probability that a household with a backyard pig farm would illegally swill-feed their swine	0.14	Schembri et al. (2010)
Dose-response coefficient (*r*)	Likelihood that a single ASF virus is able to initiate an infection in a pig through oral route	$1.21*10^{-5}$	Gale (2004)
Boar contact rate ($p_{CW'}$)	Probability that wild boar approach and try to contact landfill sites, $p_{CW'}$	0.5*179.5/370.9	Assumed same as mean for live boar contact with boar carcass – see Table 2.
Boar access to the landfill site (p_{AW})	Probability that a wild boar is able to gain access to a landfill site	0.1	Herrera-Ibatá et al. (2017)
Waste disposal (household) ($p_L(H)$)	Proportion of waste from a household will be disposed of in a landfill or other rubbish disposal that wild boar can have contact with	0.95	Herrera-Ibatá et al. (2017)
Waste disposal (restaurant) ($p_L(R)$)	Proportion of waste from a restaurant will be disposed of in a landfill or other rubbish disposal that wild boar can have contact with	0.84	Herrera-Ibatá et al. (2017)
Waste availability (T_W)	Duration of time that the waste will be available to the boar	1/365	Assumption

Supplementary Info S2: Additional Results

The probability of at least one infection in pigs for the zoomed-in regions for the wild boar movement pathway is shown in Figure 2.

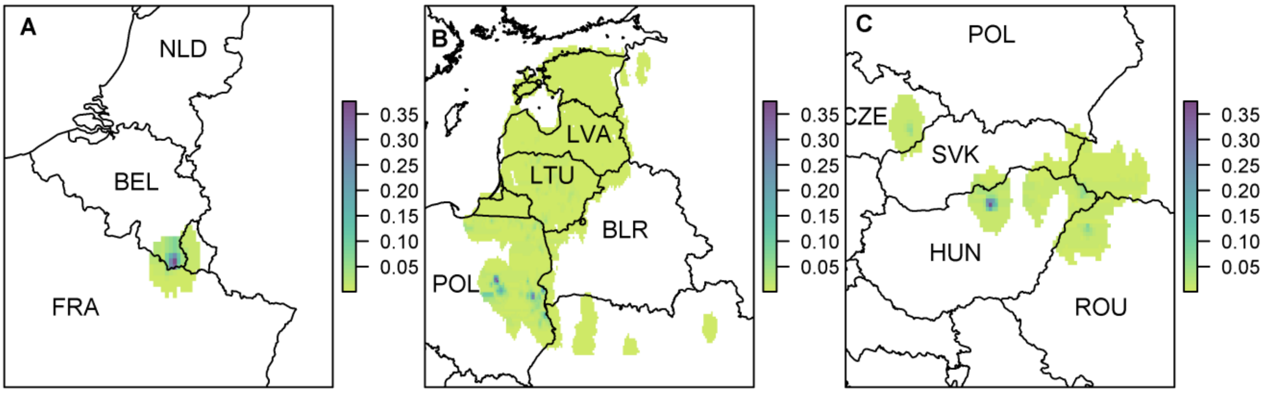

Figure 2 The probability of at least one infection in pigs with ASFV in 2019 due to the movement of wild boar in Europe plotted at a $100km^2$ cell level. We zoom in to three regions where there were cases in 2018 (A) Belgium; (B) Poland, Latvia and Lithuania; and (C) Hungary, Czech Republic and Romania. Countries are indicated by their ISO3 code.

Summary of Risk across Europe

We present summaries of the probability of infection across all cells in EU MS for both boar and pigs. These tables and histograms indicate more clearly how many cells have the lowest probability of infection versus how many have higher probability of infection. Most cells with non-zero probability of infection across Europe are in the lowest estimates of risk for each pathway and overall. The probability of infection in pigs across all $100km^2$ cells in Europe in summarised in Figure *3* and Table 6.

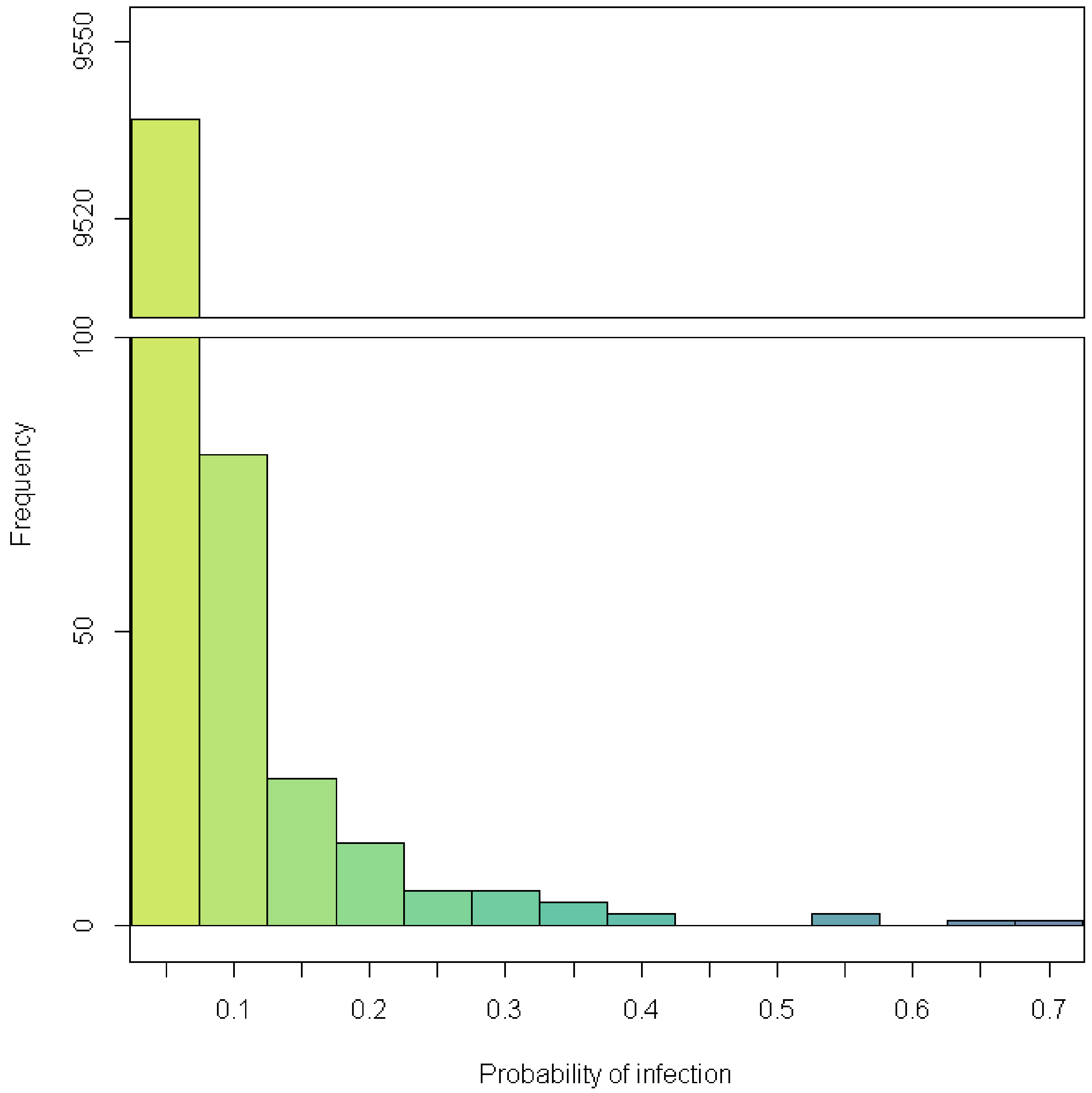

Figure 3 The frequency of 100km^2 cells with each probability of infection in pigs across Europe for all 3 pathways combined. Note the break in the y-axis since the number of cells with probability of infection between 0 – 0.05 is much higher than the number of cells with a higher probability of infection.

Table 6 The probability of infection in pigs across all EU MSs at a 100km^2 cell level, summarised as the number of cells with each probability for the 3 pathways of legal trade of live pigs, legal trade of pig meat products and wild boar movement.

Probability of Infection in Pigs					
Legal trade of live pigs		Legal trade of pig meat products		Wild boar movement	
Probability of Infection	Number of Cells	Probability of Infection	Number of Cells	Probability of Infection	Number of Cells
0 - 0.1	270	0.0001	2458	0 – 0.05	9767
0.1 – 0.2	12	0.0002	570	0.05 – 0.1	67
0.2 – 0.3	5	0.0003	131	0.1 – 0.15	18
0.3 – 0.44	2	0.0004	23	0.15 – 0.2	9
0.4 – 0.5	0	0.0005	6	0.2 – 0.25	5
0.5 – 0.6	2	0.0006	1	0.25 – 0.3	2
0.6 – 0.7	2	0.0007	1	0.3 – 0.35	3
				0.35 – 0.4	1

Similarly the probability of infection in boar is summarised across all cell in Figure 4 and Table 7.

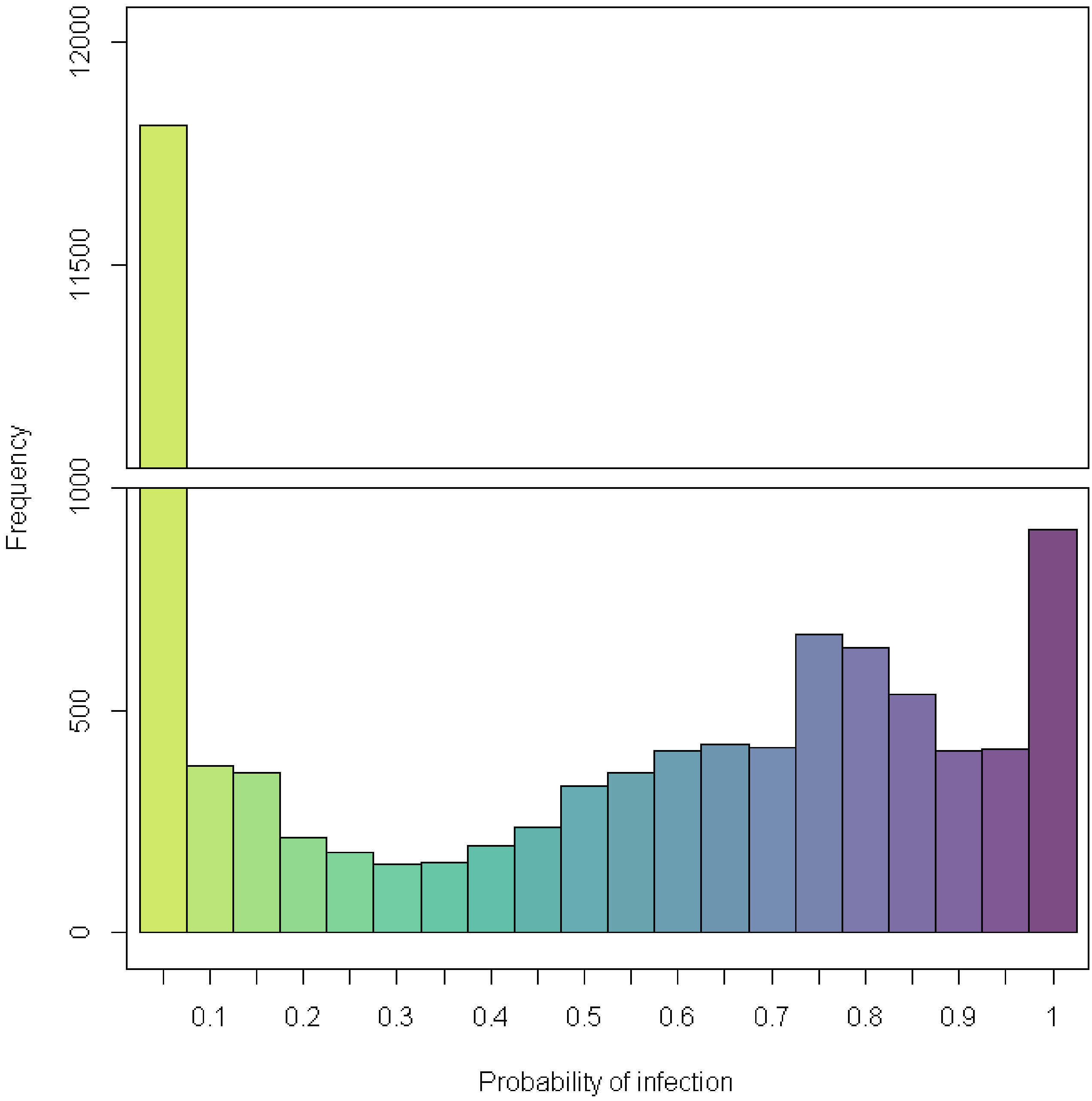

Figure 4 The frequency of 100km^2 cells with each probability of infection in boar across Europe for all 2 pathways combined. Note the break in the y-axis since the number of cells with probability of infection between 0 – 0.05 is much higher than the number of cells with a higher probability of infection.

Table 7 The probability of infection in boar across all EU MSs at a 100km^2 cell level, summarised as the number of cells with each probability for the 2 pathways of legal trade of live pigs, legal trade of pig meat products and wild boar movement.

Probability of Infection in Boar			
Legal trade of pig meat products		Wild boar movement	
Probability of Infection	Number of Cells	Probability of Infection	Number of Cells
0 – 0.01	345943	0 – 0.1	96805
0.01 – 0.02	127	0.1 – 0.2	1214
0.02 – 0.03	40	0.2 – 0.3	618
0.03 – 0.04	24	0.3 – 0.4	425
0.04 – 0.05	21	0.4 – 0.5	644
0.05 – 0.06	3	0.5 – 0.6	874
0.06 – 0.07	1	0.6 – 0.7	941
0.07 – 0.08	9	0.7 – 0.8	1456
0.08 – 0.09	2	0.8 – 0.9	1013
0.09- 0.1	92	0.9- 1	1320
0.1 – 0.11	85		

Risk for each Country

To compare which of the pathways are of the highest risk, we present the overall probability and the probability per pathway of infection in pigs in 2019 at an EU MS country level (Table 8). That is, for each country we present the probability of at least one infection in boar or pigs per pathway and through any of the 3 pathways. This indicates that for most MSs, the highest risk pathway is either the legal trade of live pigs or the movement of wild boar. The legal trade of pig meat products is rarely the highest risk, with Austria and Slovenia as the only countries indicating this (although the highest risk by this pathway is still only a 5% chance of infection in pigs). For many MSs, the movement of wild boar pathway is not applicable, as they are not geographically close enough to any reported cases. When it is applicable, more than half the time this is the highest risk pathway for those countries. Western European countries mostly have legal trade of live pigs as the highest risk pathway.

Table 8 The probability of at least one infection with ASFV in pigs in each country for all pathways combined and for the individual pathways. Countries are listed from greatest overall risk to lowest. The pathways are colored according to the risk per pathway – the darker the color of pathway for that country, the riskier for that country. Countries are indicated by their ISO3 code.

Country	Overall Risk	Legal trade in live pigs	Legal trade in pig meat products	Movement of wild boar
POL	1	0.994384	0.003195	1
LTU	0.999619	0.975893	0.072077	0.951278
ROU	0.892792	0.015095	0.083603	0.801402
HUN	0.877448	0.150422	0.144967	0.771641
LVA	0.750279	0.243421	0.02352	0.45989
EST	0.522105	0.0002	0.002198	0.368578
CZE	0.307924	1.00E-04	0.0003	0.237292
SVK	0.233413	0.010486	0.0002	0.024306
ITA	0.191544	0.007873	0	0.1534
BGR	0.133966	0.002199	0.018331	0.071107
BEL	0.129847	0.0008	0.0002	0.093985
DEU	0.07141	0.069458	0.0003	0
FRA	0.066802	0.015407	0	0.007274
AUT	0.055893	0.006093	0.051908	0
NLD	0.051984	0.051889	0	0
LUX	0.028322	0.002697	0	0.009755
HRV	0.006083	0.003894	0	0
SVN	0.00449	0.0004	0.003693	0
PRT	0.002999	0.002899	1.00E-04	0
ESP	0.002198	0.001899	0.0003	0
GRC	0.001699	0.0013	0.0004	0
CYP	0	0	0	0
DNK	0	0	0	0
FIN	0	0	0	0
GBR	0	0	0	0
IRL	0	0	0	0
SWE	0	0	0	0

The overall risk to boars by country is found in Table 9. As trade in live pigs does not include a transmission to boar component, only the two other pathways are combined to produce the overall risk. For all the highest risk countries the movement of wild boar is the pathway with greatest risk, although many of these high risk countries have a high probability for both pathways. For all other countries, the legal trade of pig meat products is highest. This is because the movement of wild boar pathway can only affect those countries with wild boar cases already or neighbouring those that do, whereas the legal trade of pig meat products can affect all countries that have wild boar.

Table 9 The probability of at least one infection with ASFV in boar in each country for all pathways combined and for the individual pathways (trade in live pigs not included as boar cannot be infected via this route). Countries are listed from greatest overall risk to lowest. The individual pathways are colored according to the risk per pathway – the darker the color of pathway for that country, the riskier for that country. Countries are indicated by their ISO3 code.

Country	Overall Risk	Legal trade in pig meat products	Movement of wild boar
ITA	1	0.996513	1
EST	1	0.963429	1
HUN	1	0.893238	1
LVA	1	0.878729	1
LTU	1	0.851094	1
FRA	1	0.641203	1
POL	1	0.505774	1
BGR	1	0.376832	1
CZE	1	0.370403	1
ROU	1	0.197674	1
BEL	1	0.097364	1
LUX	1	0.064819	1
SVK	1	0.076066	0.989928
HRV	0.993581	0.995174	0
AUT	0.973209	0.979067	0
GRC	0.933435	0.933435	0
SWE	0.853432	0.853432	0
DEU	0.640135	0.637969	0
DNK	0.571805	0.571805	0
PRT	0.493721	0.493721	0
ESP	0.421196	0.621301	0
GBR	0.330601	0.330601	0
SVN	0.083725	0.083725	0
NLD	0.048971	0.048971	0
FIN	0.0002	0.0002	0
CYP	0	0	0
IRL	0	0	0
MLT	0	0	0
MNE	0	0	0
TUR	0	0	0

Supplementary Info S3: Scenario and Sensitivity Analysis

Scenario Analysis

Methods

We perform a scenario analysis for this pathway to investigate the role of detection of ASF-infected animals at entry to the country. In the baseline results we assume that no detection takes place, because all testing of pigs or pig meat in the EU is voluntary. However, it is possible that some countries implement testing of animals on entry, perhaps by observation or by clinical tests. To explore the effect that successful detection of animals would have on the probability of infection with ASF in pigs across Europe, we perform a scenario in which all countries have a probability of 0.375 of detecting each infected pig. This value of 0.375 is chosen based upon the assumption that most detection would be via visual inspection and then comparing the length of the latent period in pigs compared to the length of time of clinical signs. We use a value of 10 days for the number of days before clinical signs appear (see Table 5) and a value of 6 days for the number of days that clinical signs are apparent (the mode of the infectious period, Table 1). Thus the proportion of time that animals show clinical signs compared to the overall time that they are infectious is 6/(6+10) = 0.375. To implement this into our risk assessment framework, we set $J(c)$ to be the number of live pigs successfully passing inspection and entering each cell c, and is calculated as follows:

$$J(c) \sim Bin(I(c), p_D),$$

where $I(c)$ is the number of infected animals entering cell c prior to any inspection (as per the main text) and p_D is the probability of detecting infection in each pig.

Results

The results for the scenario analysis for the legal trade of live pig pathway, in which we assume that there is a probability of detection of 0.375, are presented in Figure 5. The implementation of detection results in approximately the same number of farms across Europe with non-negligible risk (a decrease of 0.2% in the number of farms). The farm with the maximum probability of infection in pigs in the baseline scenario is 0.652 whereas in the scenario it has a probability of infection of 0.482, a reduction of 26%. The mean probability of infection on a farm is 0.023 in the baseline results whereas it is 0.018 in the scenario, a reduction of 22%. Furthermore, some farms do not experience any reduction in risk. Therefore, there is a non-linear relationship between the probability of detection and the probability of infection on a farm, such that the probability of detection needs to be higher than the desired reduction in risk on a farm, although it does seem to reduce the risk most for the highest risk farms.

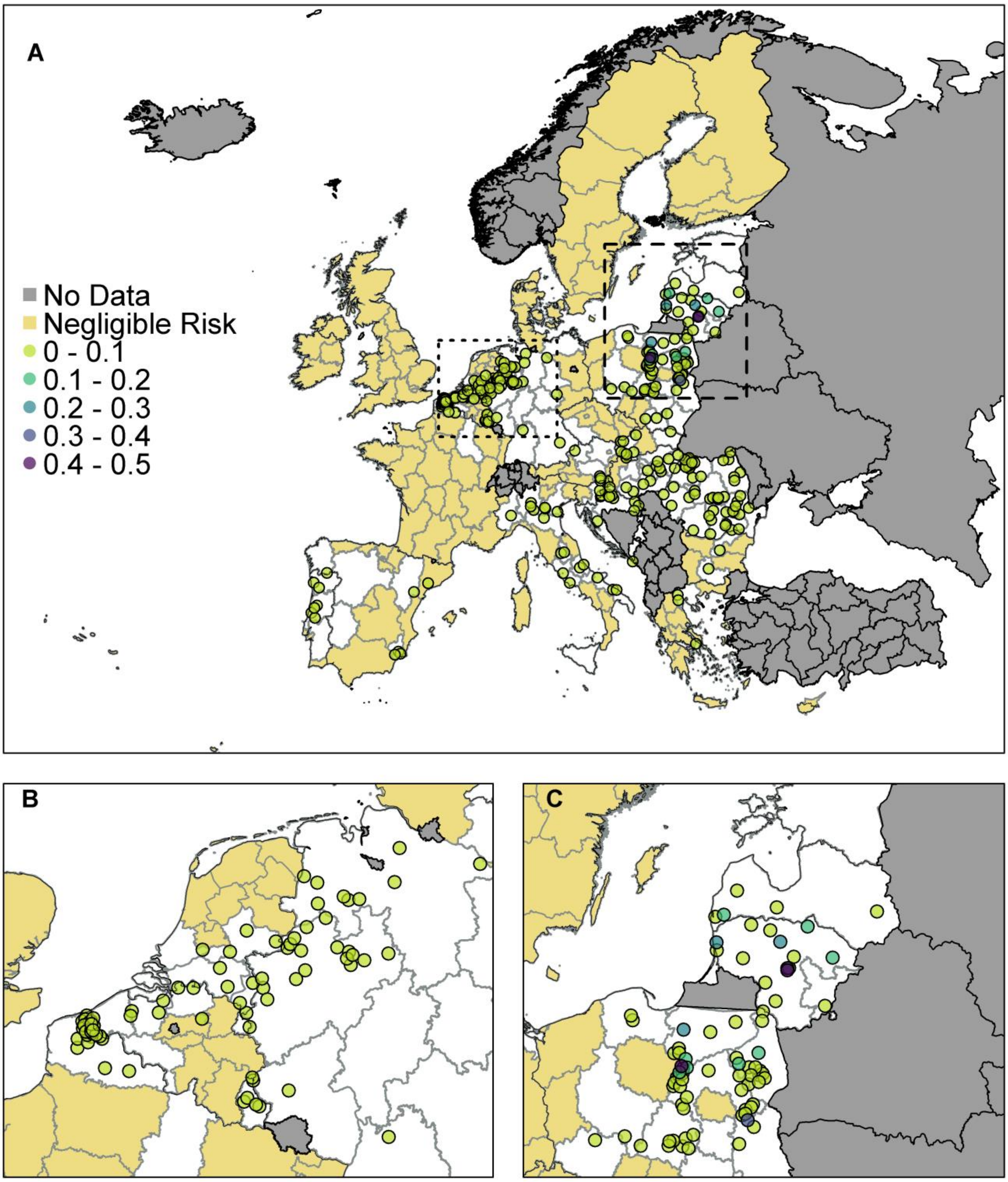

Figure 5 The probability of at least one infection of ASFV in pigs in 2019 from trade of live pigs at a farm level assuming a probability of detection of 0.375. In (A) all of Europe is plotted while in (B) the map is zoomed in to the dotted rectangle in (A) and in (C) the map is zoomed in to the dashed rectangle in (A). All farms indicated by a circle imported at least one infected animal in at least one simulation and the color indicates the probability that one or more susceptible pigs became infected. Countries in grey have insufficient data to complete the risk assessment. All farms in the regions with negligible risk either did not import any pigs or did not import any infected pigs. © EuroGeographics for the administrative boundaries

Sensitivity Analysis

Methods

Due to the most uncertainty appearing in the legal trade of food pathway, and since uncertainty in the legal trade of animals and movement of wild boar pathways was explored in Taylor et al. (2019a) and Taylor et al. (2019b) respectively, we consider only parameters in the food pathway in the sensitivity analysis. The parameters we consider and their values within the sensitivity analysis are provided in Table *10*. We do not explore all parameters in this pathway but focus on those which are most uncertain.

Table 10 The parameters we explore in the sensitivity analysis along with their original value in the baseline results and the value used in the sensitivity analysis.

Parameter	Original Value	Value used in sensitivity analysis	Reasoning
Probability food is not cooked to at least 60°C ($p_{C<60}$)	0.2	0.32	Based on a bootstrap analysis of the EcoSure (2008) data, a maximum of 32% of food is not cooked to at least 60°C
Proportion of food wasted at a restaurant ($p_W(R)$) or household ($p_W(H)$)	Normal(μ, 6.6)<br>Where μ is:<br>UK: 0.23<br>Netherlands: 0.08<br>Denmark: 0.07<br>Germany: 0.13<br>Other EU MSs: 0.145	For all EU MS:<br>Normal(0.23, 6.6)	We set all EU MS to be the highest estimate found in literature
Probability of illegal swill-feeding (p_{SF})	0.14	0.22	Maximum value found in literature (Hernández-Jover et al. 2016)
Probability boar can access the landfill site (p_{AW})	0.1	0.2	Use the maximum from the pert distribution pert(0.05, 0.1, 0.2) used by Herrera-Ibatá et al. (2017)
Duration of waste availability (T_W)	1/365	7/365	An assumption that waste may be available for up to a week

We calculate the probability of at least one infection in boar and pigs in each 100km^2 cell similar to the baseline results. We then compare the results using the changed parameters with the baseline results to analyse which parameters the food pathway is most sensitive to. The maps are the best way to get a good overview of the effect of the uncertain parameters on the model, and so these are provided in Supplementary Info S2. However, in order to compare across parameters to see which the model is most senstive to, we use two metrics. We focus on the cells which we define to be a hotspot in order to see how much the model affects this aspect of the results. We set a relatively low bound for the definition of a hotspot – a probability of infection of >0.02 for wild boar and a probability of infection of >0.0001 for pigs. Our first metric is the number of hotspot cells that exist

across Europe while the second the is the distribution of the probability of infection for hotspot cells in Europe, both calculated separately for boar and pigs.

Additional Results

We provide for further clarification the maps of the 5 sensitivity analysis for the food pathway.

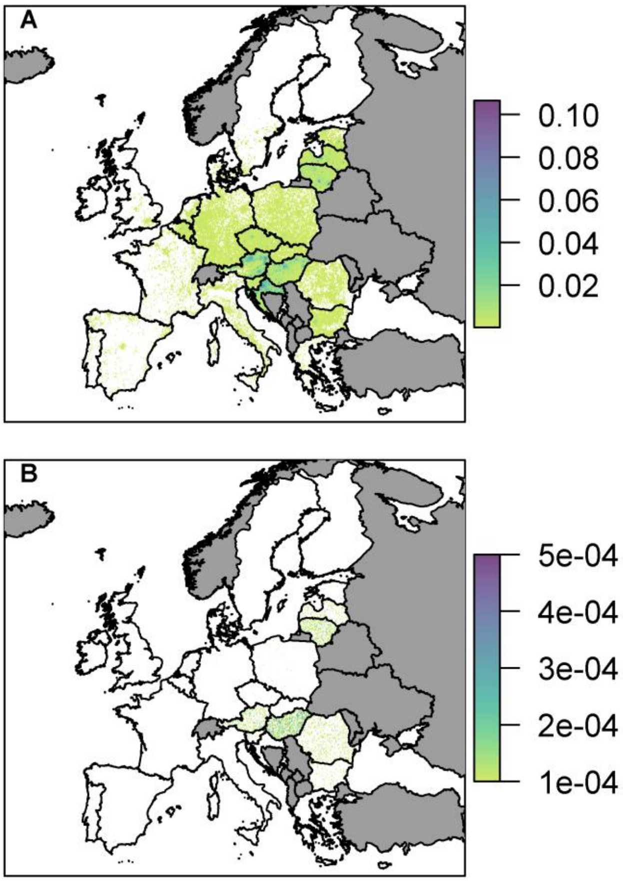

Figure 6 The probability of at least one infection of ASFV in 2019 in (A) wild boar and (B) pigs, via trade in legal pig meat products, plotted at a 100km^2 cell level across Europe for sensitivity WA - duration of waste is increased from 1 day to 7 days. Countries in grey have insufficient data to complete the risk assessment.

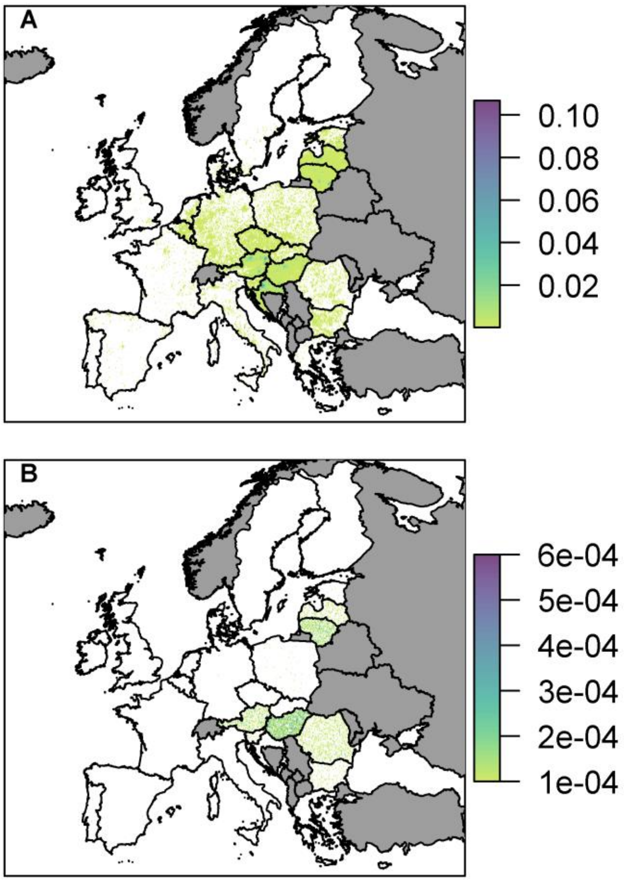

Figure 7 The probability of at least one infection of ASFV in 2019 in (A) wild boar and (B) pigs, via trade in legal pig meat products, plotted at a $100km^2$ cell level across Europe for sensitivity WP – the proportion of meat products that go to waste in a household or restaurant is increased. Countries in grey have insufficient data to complete the risk assessment.

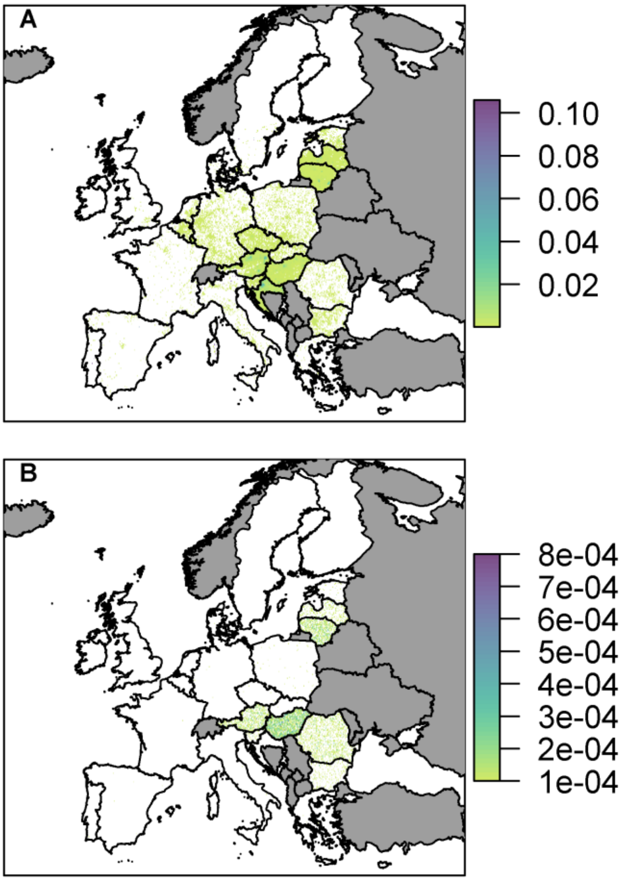

Figure 8 The probability of at least one infection of ASFV in 2019 in (A) wild boar and (B) pigs, via trade in legal pig meat products, plotted at a 100km^2 cell level across Europe for sensitivity SF – the probability of illegal swill-feeding on a backyard farm is increased from 0.14 to 0.22. Countries in grey have insufficient data to complete the risk assessment.

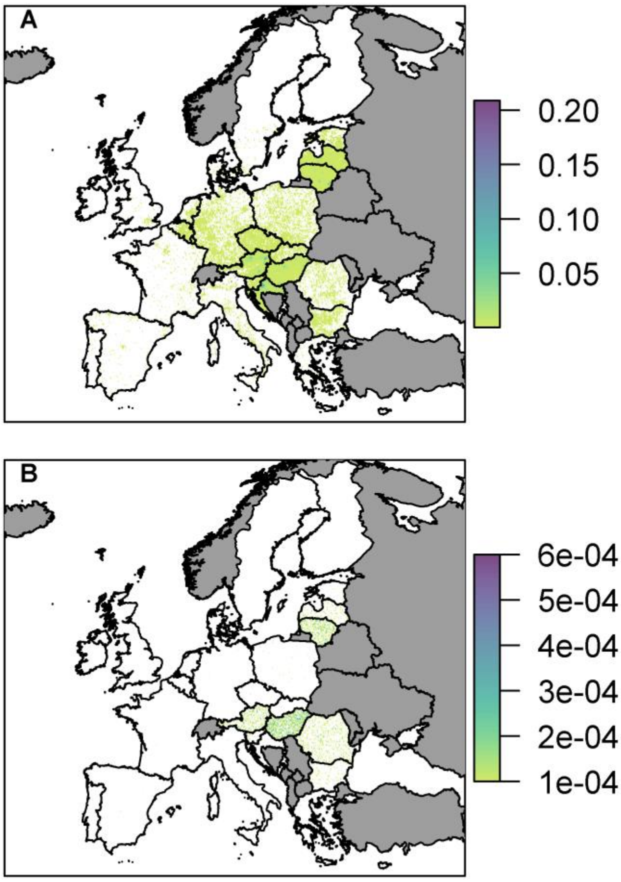

Figure 9 The probability of at least one infection of ASFV in 2019 in (A) wild boar and (B) pigs, via trade in legal pig meat products, plotted at a 100km^2 cell level across Europe for sensitivity BA – the probability that boar are able to access a waste site is increased from 0.1 to 0.2. Countries in grey have insufficient data to complete the risk assessment.

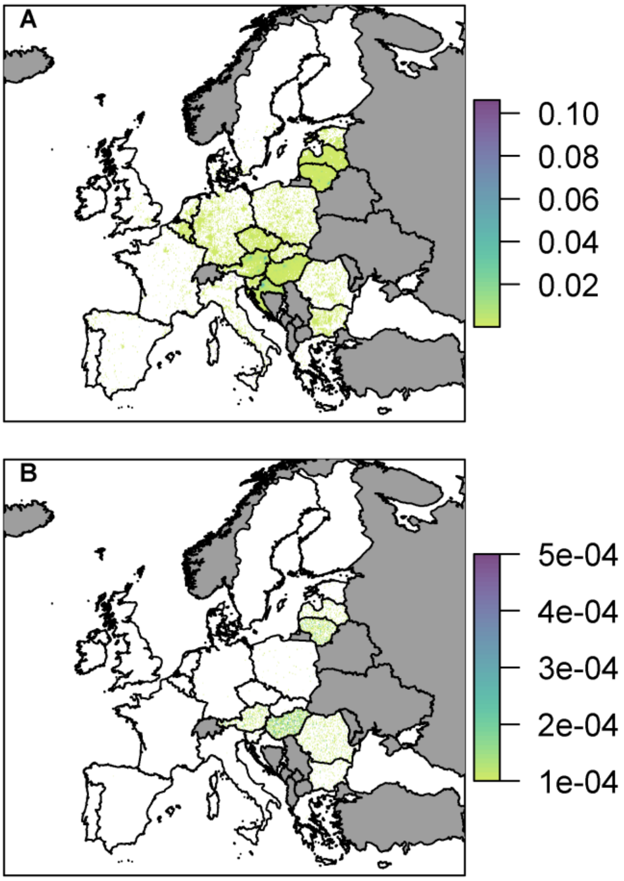

Figure 10 The probability of at least one infection of ASFV in 2019 in (A) wild boar and (B) pigs, via trade in legal pig meat products, plotted at a 100km^2 cell level across Europe for sensitivity FC – the probability that food is not cooked sufficiently to kill the virus is increased from 0.2 to 0.32. Countries in grey have insufficient data to complete the risk assessment.

References

Adkin, A., H. Coburn, T. England, S. Hall, E. Hartnett, C. Marooney, M. Wooldridge, E. Watson, J. Cooper and T. Cox (2004). "Risk assessment for the illegal import of contaminated meat and meat products into Great Britain and the subsequent exposure of GB livestock (IIRA): foot and mouth disease (FMD), classical swine fever (CSF), African swine fever (ASF), swine vesicular disease (SVD)." Veterinary Laboratories Agency, New Haw.

Arias, M., C. Jurado, C. Gallardo, J. Fernández-Pinero and J. Sánchez-Vizcaíno (2018). "Gaps in African swine fever: Analysis and priorities." Transboundary and emerging diseases **65**: 235-247.

Badminton Feeds. (2019). "Advice."   Retrieved 2019, June, from http://www.badmintonfeeds.co.uk/advice:6.htm.

Blackwell, J. H. (1984). "Foreign animal disease agent survival in animal products: recent developments." Journal of the American Veterinary Medical Association **184**(6): 674-679.

Blome, S., C. Gabriel and M. Beer (2013). "Pathogenesis of African swine fever in domestic pigs and European wild boar." Virus Research **173**(1): 122-130.

Blome, S., C. Gabriel, K. Dietze, A. Breithaupt and M. Beer (2012). "High virulence of African swine fever virus caucasus isolate in European wild boars of all ages." Emerging infectious diseases **18**(4): 708.

Chenais, E., K. Depner, V. Guberti, K. Dietze, A. Viltrop and K. Ståhl (2019). "Epidemiological considerations on African swine fever in Europe 2014–2018." Porcine Health Management **5**(1): 6.

EcoSure (2008). 2007 U.S. Cold Temperature Evaluation. **2016**.

Eurostat. (2017). "Eurostat Bulk Download Listing."   Retrieved 18th Oct, 2018, from http://ec.europa.eu/eurostat/estat-navtree-portlet-prod/BulkDownloadListing.
FAO (2011). Global food losses and food waste – Extent, causes and prevention. Rome.
Gabriel, C., S. Blome, A. Malogolovkin, S. Parilov, D. Kolbasov, J. P. Teifke and M. Beer (2011). "Characterization of African swine fever virus Caucasus isolate in European wild boars." Emerging infectious diseases **17**(12): 2342.
Gale, P. (2004). "Risks to farm animals from pathogens in composted catering waste containing meat." Veterinary Record **155**(3): 77-82.
Guinat, C., S. Gubbins, T. Vergne, J. Gonzales, L. Dixon and D. Pfeiffer (2016). "Experimental pig-to-pig transmission dynamics for African swine fever virus, Georgia 2007/1 strain." Epidemiology & Infection **144**(1): 25-34.
Guinat, C., A. L. Reis, C. L. Netherton, L. Goatley, D. U. Pfeiffer and L. Dixon (2014). "Dynamics of African swine fever virus shedding and excretion in domestic pigs infected by intramuscular inoculation and contact transmission." Veterinary research **45**(1): 93.
Gulenkin, V. M., F. I. Korennoy, A. K. Karaulov and S. A. Dudnikov (2011). "Cartographical analysis of African swine fever outbreaks in the territory of the Russian Federation and computer modeling of the basic reproduction ratio." Preventive Veterinary Medicine **102**(3): 167-174.
Hartnett, M., A. Adkin, T. England, H. Coburn, E. Watson, S. Hall, C. Marooney and M. Wooldridge (2014). Risk Assessment for the Import of Contaminated Meat and Meat Products into Great Britain and the Subsequent Exposure of GB Livestock. **2019**.
Hernández-Jover, M., N. Schembri, P. K. Holyoake, J. A. L. M. L. Toribio and P. A. J. Martin (2016). "A comparative assessment of the risks of introduction and spread of foot-and-mouth disease among different pig sectors in Australia." Frontiers in Veterinary Science **3**(SEP).
Herrera-Ibatá, D. M., B. Martínez-López, D. Quijada, K. Burton and L. Mur (2017). "Quantitative approach for the risk assessment of African swine fever and Classical swine fever introduction into the United States through legal imports of pigs and swine products." PLoS ONE **12**(8).
Keuling, O., K. Lauterbach, N. Stier and M. Roth (2010). "Hunter feedback of individually marked wild boar Sus scrofa L.: dispersal and efficiency of hunting in northeastern Germany." European Journal of Wildlife Research **56**(2): 159-167.
Kleiboeker, S. B. (2002). "Swine fever: classical swine fever and African swine fever." Veterinary Clinics of North America - Food Animal Practice **18**(3): 431-451.
Kovalenko, Y. R. (1964). "Viability of ASF in the environment." Vestnik Sel' Skokhozyaistrenoi Nanki **9**: 62.
Leaper, R., G. Massei, M. Gorman and R. Aspinall (1999). "The feasibility of reintroducing wild boar (Sus scrofa) to Scotland." Mammal Review **29**(4): 239-258.
Massei, G., P. Genov, B. Staines and M. Gorman (1997). "Factors influencing home range and activity of wild boar (Sus scrofa) in a Mediterranean coastal area." Journal of Zoology **242**(3): 411-423.
McKercher, P. D. (1987). "Survival of viruses in "Prosciutto di Parma" (Parma Ham)." Canadian Institute of Food Science & Technology **20**: 267.
McKercher, P. D., W. R. Hess and F. Hamdy (1978). "Residual viruses in pork products." Applied and Environmental Microbiology **35**(1): 142-145.
Mebus, C., M. Arias, J. M. Pineda, J. Tapiador, C. House and J. M. Sánchez-Vizcaíno (1997). "Survival of several porcine viruses in different Spanish dry cured meat products." Food Chemistry **59**(4): 555-559.
Morley, R. (1993). "A model for the assessment of the animal disease risks associated with the importation of animals and animal products." REVUE SCIENTIFIQUE ET TECHNIQUE-OFFICE INTERNATIONAL DES EPIZOOTIES **12**: 1055-1055.
Olesen, A., L. Lohse, A. Boklund, T. Halasa, G. Belsham, T. Rasmussen and A. Bøtner (2018). "Short time window for transmissibility of African swine fever virus from a contaminated environment." Transboundary and emerging diseases.
Pietschmann, J., C. Guinat, M. Beer, V. Pronin, K. Tauscher, A. Petrov, G. Keil and S. Blome (2015). "Course and transmission characteristics of oral low-dose infection of domestic pigs and European wild boar with a Caucasian African swine fever virus isolate." Archives of virology **160**(7): 1657-1667.
Podgórski, T., M. Apollonio and O. Keuling (2018). "Contact rates in wild boar populations: Implications for disease transmission." The Journal of Wildlife Management **82**(6): 1210-1218.

Podgórski, T., M. Scandura and B. Jędrzejewska (2014). "Next of kin next door–philopatry and socio-genetic population structure in wild boar." Journal of Zoology **294**(3): 190-197.
Probst, C., A. Globig, B. Knoll, F. J. Conraths and K. Depner (2017). "Behaviour of free ranging wild boar towards their dead fellows: potential implications for the transmission of African swine fever." Royal Society open science **4**(5): 170054.
Sànchez-Vizcaíno, J. M., L. Mur, A. D. S. Bastos and M. L. Penrith (2015). "New insights into the role of ticks in African swine fever epidemiology." OIE Revue Scientifique et Technique **34**(2): 503-511.
Schembri, N., M. Hernández-Jover, J. A. Toribio and P. K. Holyoake (2010). "Feeding of prohibited substances (swill) to pigs in Australia." Australian Veterinary Journal **88**(8): 294-300.
Simons, R. R., V. Horigan, P. Gale, R. D. Kosmider, A. C. Breed and E. L. Snary (2016). "A generic quantitative risk assessment framework for the entry of bat-borne zoonotic viruses into the European Union." PloS one **11**(10): e0165383.
Taylor, R. A., A. D. Berriman, P. Gale, L. A. Kelly and E. L. Snary (2019a). "A generic framework for spatial quantitative risk assessments of infectious diseases: Lumpy skin disease case study." Transboundary and emerging diseases **66**(1): 131-143.
Taylor, R. A., T. Podgórski, R. R. L. Simons, S. Ip, P. Gale, L. A. Kelly and E. L. Snary (2019b). "Predicting spread and effective control measures for African swine fever– should we blame the boars?" bioRxiv: 654160.
Temple, D., X. Manteca, A. Velarde and A. Dalmau (2011). "Assessment of animal welfare through behavioural parameters in Iberian pigs in intensive and extensive conditions." Applied Animal Behaviour Science **131**(1-2): 29-39.
Thulke, H. H. and M. Lange (2017). "Simulation-based investigation of ASF spread and control in wildlife without consideration of human non-compliance to biosecurity." EFSA Supporting Publications **14**(11).
Truvé, J. and J. Lemel (2003). "Timing and distance of natal dispersal for wild boar Sus scrofa in Sweden." Wildlife Biology **9**(SUPPL 1): 51-57.
Vanham, D., F. Bouraoui, A. Leip, B. Grizzetti and G. Bidoglio (2015). "Lost water and nitrogen resources due to EU consumer food waste." Environmental Research Letters **10**(8).
WRAP (2008). The food we waste. www.wrap.org.uk.
